# Induced degradation of Ufd1 reveals regulation of cohesin by the VCP/p97^Ufd1-Npl4^ complex

**DOI:** 10.26508/lsa.202603698

**Published:** 2026-09-03

**Authors:** Daniella Munro, Maike Reinders, Aikaterini Pravi, Johannes van den Boom, Pinki Gahlot, Ekaterina Isaakova, Caio AB Oliveira, Dominik Boos, Petra Beli, Hemmo Meyer

**Affiliations:** 1 Molecular Biology I, Center of Medical Biotechnology, Faculty of Biology, University of Duisburg-Essen , Essen, Germany; 2 Institute of Molecular Biology (IMB) , Mainz, Germany; 3 Molecular Genetics II, Center of Medical Biotechnology, Faculty of Biology, University of Duisburg-Essen , Essen, Germany; 4 Institute of Developmental Biology and Neurobiology (IDN), Johannes Gutenberg-Universität, Mainz, Germany

## Abstract

The AAA ATPase VCP/p97 has emerged as a critical regulator of ubiquitin and chromatin-associated processes but progress in understanding has been hampered by the complexity of p97 functions and the various p97 cofactors involved. Here, we combined ubiquitin profiling with acutely induced degradation of the Ufd1 subunit of the p97 ubiquitin adapter, Ufd1-Npl4, in human cells. We identified a set of chromatin regulators, HUS1, XRCC1, MORF4L1, and the cohesin subunit RAD21 as targets of p97^Ufd1-Npl4^. We find that RAD21 is ubiquitylated and targeted by p97^Ufd1-Npl4^ specifically in S phase to remove a subpopulation of cohesin from chromatin. Acute degradation of Ufd1 in S phase, after replication licensing is completed, impedes replication and leads to replication-associated DNA damage. Our findings suggest that a fraction of cohesin rings need to be removed by p97^Ufd1-Npl4^ from DNA to allow unhindered replication and reveal a critical function of p97 that ensures genome stability.

## Abstract (precis)

The p97-Ufd1-Npl4 complex targets ubiquitylated RAD21 to extract a subpopulation of cohesion specifically in S-phase to facilitate DNA replication and suppress replication-associated DNA damage.

## Introduction

The essential AAA ATPase p97 (also called VCP for Valosin-Containing Protein, or Cdc48 in yeast) functions in a myriad of processes with hundreds of substrate proteins to ensure cellular homeostasis and genome stability, and pharmacological inhibition of p97 is considered a possible strategy for cancer treatment (Anderson et al, 2015; Ye et al, 2017; van den Boom & Meyer, 2018). Mechanistically, p97 uses the energy of ATP hydrolysis to unfold substrate proteins or to extract proteins from binding partners and structures such as chromatin (Ye et al, 2017). Substrate targeting can occur in a ubiquitin-dependent or ubiquitin-independent manner and is mediated by distinct sets of p97 adapter proteins and accessory factors (Buchberger et al, 2015; Braxton & Southworth, 2023; Meyer & van den Boom, 2023). The major adapter for ubiquitin-labeled substrates is the Ufd1-Npl4 heterodimer (Meyer et al, 2002). Ufd1-Npl4 binds the conjugated ubiquitin chain and funnels ubiquitin and the attached substrate into central pore of p97 thus initiating the threading mechanism that triggers substrate protein unfolding or extraction (Twomey et al, 2019; Williams et al, 2023). Because p97^Ufd1-Npl4^ acts downstream of substrate ubiquitylation, inactivation of p97 or Ufd1-Npl4 leads to accumulation of ubiquitylated substrates (Wojcik et al, 2004).

The p97^Ufd1-Npl4^ complex has been linked to several chromatin-associated processes (Franz et al, 2016a). Recurringly, p97 has been seen to extract specific ubiquitin-labeled substrates from chromatin at critical time points to orchestrate processes such as DNA damage repair and replication. Although the range of chromatin-associated functions of p97^Ufd1-Npl4^ is expected to be much wider, only a number of substrates have been identified (Heidelberger et al, 2018). A difficulty in uncovering additional targets of p97^Ufd1-Npl4^ is that p97 inhibitors quickly induce strong cytopathic effects, whereas slow and long-term depletion of p97 or its adapters causes pleotropic effects and adaptations that confound downstream data interpretation.

For a more targeted approach to p97^Ufd1-Npl4^, we therefore applied rapid induced-degradation of the adapter. We targeted Ufd1 because Ufd1 and Npl4 function as an adapter only when present together, and Npl4 fails to bind to p97 in the absence of Ufd1 (Meyer et al, 2000). By combining rapid induced-degradation of Ufd1 with ubiquitin profiling, we identified a set of chromatin regulators including RAD21 (also called Scc1) as p97^Ufd1-Npl4^ substrates. RAD21 is the kleisin subunit that closes the cohesin ring that consists of SMC1 and SMC3, as well as alternatively associated factors STAG1 or STAG2 (Nasmyth & Haering, 2009). Cohesin encircles DNA and thus exerts critical functions in chromatin organization, DNA repair and sister chromatid cohesion (Uhlmann, 2025). Functional analysis in this study reveals that p97^Ufd1-Npl4^, by targeting RAD21, extracts a subpopulation of cohesin from DNA specifically during S-phase, removing hurdles for replication and safeguarding against replication-associated DNA damage.

## Results

### Ubiquitin-profiling upon rapid Ufd1 degradation identifies chromatin-associated targets of p97^Ufd1-Npl4^

**Figure 1.**
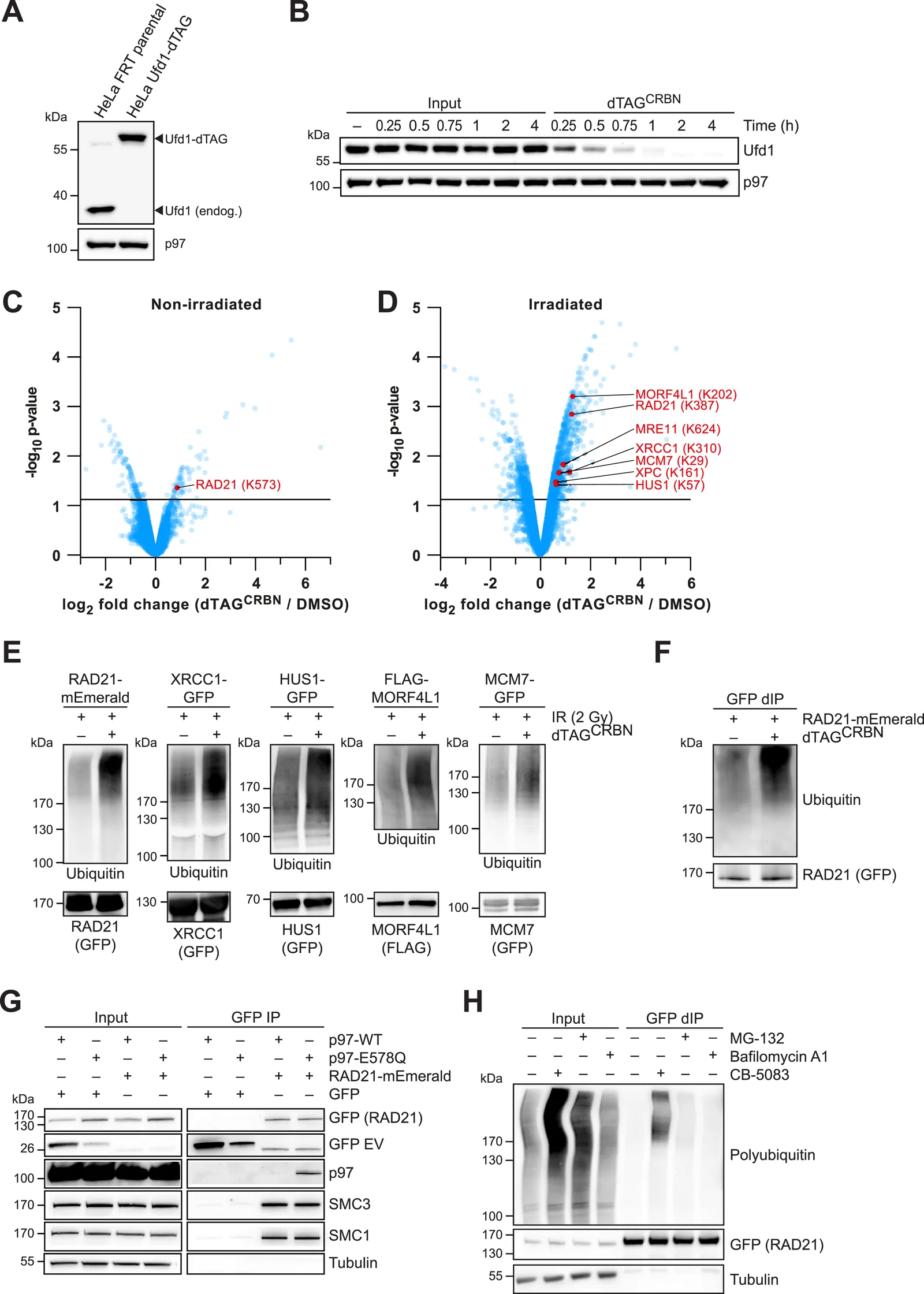
Ubiquitin-profiling upon Ufd1 degradation identifies chromatin-associated targets of p97^Ufd1-Npl4^. **(A)** A transgene coding for FKBP12^F36V^-tagged Ufd1 was introduced into HeLa cells as the only source of Ufd1 after knockout of the UFD1 gene. Western blot of Ufd1^FKBP12^ and parental cell lysates. **(B)** Ufd1^FKBP12^ is rapidly degraded after treatment with dTAG^CRBN^ (1 µM). Western blot of lysates at indicated time points. **(C, D)** Ubiquitin profiling using mass spectrometry of diGly remnants isolated from Ufd1^FKBP12^ cell lysates comparing control with dTAG treatments (1 µM). **(C)** Analysis of unchallenged cells (−IR, 8 h dTAG treatment) or (D) cells challenged with ionizing radiation (+IR, 1 h dTAG pretreatment, continued for 3 h after 2 Gy IR). **(E, F)** Western blot validation of ubiquitylation of indicated candidates after denaturing immunoprecipitation from control or dTAG-treated cells. See Fig S1D for gels with all controls. **(G)** RAD21 co-immunoprecipitation from cells stably expressing p97 wild type or the substrate-trapping mutant p97-E578Q transiently transfected with RAD21-mEmerald or GFP empty vector. Note increased interaction of RAD21 with p97-E578Q. **(H)** Western blot of RAD21 isolated under denaturing conditions from cells treated as indicated. Note accumulation of ubiquitylated RAD21 upon p97 inhibition (CB-5083; 5 µM, 4 h) but not proteasome inhibition (MG132; 20 µM, 4 h) or the lysosome inhibition (bafilomycin A1; 100 nM, 4 h).

**Figure S1.**
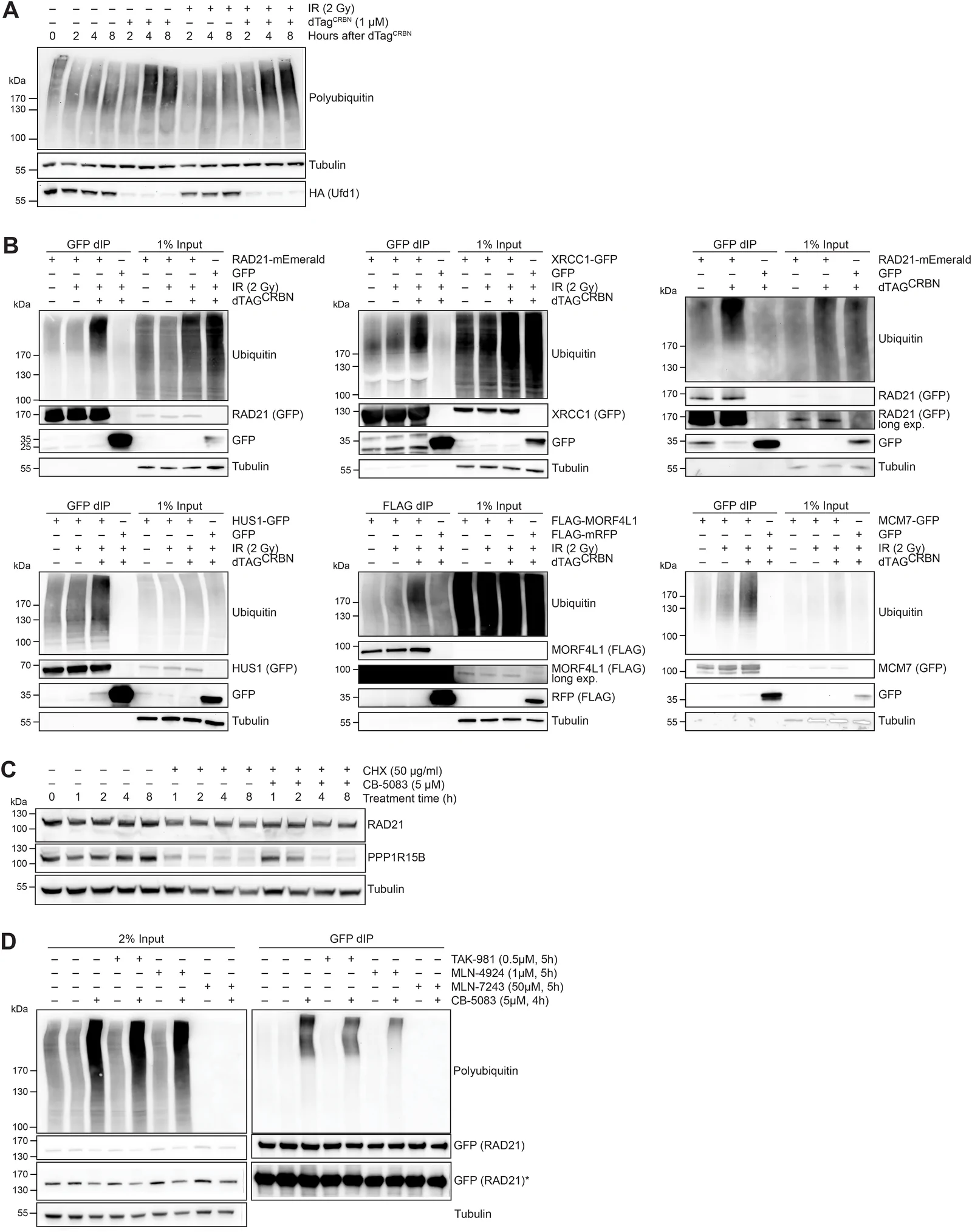
Related to Fig 1. **(A)** Western blot analysis of lysates from Ufd1^FKBP12^ cells treated with dTAG or vehicle alone for indicated time and exposed to control conditions or ionizing radiation (2 Gy). Note accumulation of ubiquitin conjugates upon induced degradation of Ufd1. **(B)** Whole Western blots for ubiquitylation validation shown in Fig 1E and F. **(C)** Cycloheximide (CHX) chase assay. Samples were taken at progressive time points after indicated treatments. Note stability of RAD21 that is not affected by CB-5083, in contrast to the unstable p97 substrate, PPP1R15B, as positive control. **(D)** Effect of inhibitors of ubiquitin (MLN7243), Nedd8 (MLN4924) and SUMO (TAK981) E1 activating enzymes on accumulation of ubiquitylated RAD21 in CB-5083-treated cells. Cells were pre-treated with E1 inhibitors for 1 h before combined treatment with CB-5083 for 4 h. Asterisk indicates longer exposure.

For an induced-degradation approach to Ufd1, we aimed to tag Ufd1 with the FKBP12^F36V^ moiety in HeLa cells to allow targeting with the dTAG^CRBN^ degrader, which links the FKBP12^F36V^ -tag with the CRBN ubiquitin ligase subunit and triggers ubiquitylation (Nabet et al, 2018). Direct tagging of the essential *UFD1L* gene that codes for Ufd1 failed. We therefore first stably introduced a transgene coding for Ufd1-FKBP12^F36V^ in HeLa cells and subsequently knocked out the Ufd1 gene so that the transgene was the only source of Ufd1 (Fig 1A). Treatment of Ufd1^FKBP12^ cells with dTAG^CRBN^ resulted in rapid degradation of Ufd1 within 1 h (Fig 1B). Generally, p97^Ufd1-Npl4^ acts downstream of substrate ubiquitylation and allows subsequent degradation or deubiquitylation. Consistent with that, induced degradation of Ufd1 caused accumulation of ubiquitin conjugates, serving as biomarker (Fig S1A). To identify possible targets of p97^Ufd1-Npl4^, we next conducted a quantitative mass spectrometry screen of ubiquitylated peptides comparing Ufd1^FKBP12^ cells treated with either vehicle alone or dTAG^CRBN^. The comparison was carried out in parallel in unchallenged cells as well as in cells subjected to ionizing radiation (IR, 2 Gy) to also probe for p97^Ufd1-Npl4^ targets specifically in the DNA damage response. Lysates were processed for ubiquitin remnant identification by mass spectrometry scoring candidates with differential ubiquitylation pattern upon Ufd1 degradation (Fig 1C and D). Among the range of proteins that accumulated in Ufd1-deficient cells upon IR compared with control IR-treated cells, we identified a series of established regulators of DNA damage repair and replication (Fig 1D). They included MCM7, MRE11 and XPC that had previously been connected to p97 (Maric et al, 2014; Moreno et al, 2014; Puumalainen et al, 2014; Kilgas et al, 2021). We confirmed accumulation of ubiquitylated MCM7 by denaturing immunoprecipitation and Western blot analysis, validating our approach (Figs 1E and S1B). In addition, we identified RAD21, HUS1, XRCC1 and MORF4L1 to accumulate in ubiquitylated form in Ufd1-deficient cells, which we confirmed by Western blot (Figs 1E and S1B). We decided to focus on RAD21, because ubiquitylated RAD21 also accumulated in unchallenged cells upon Ufd1 degradation (Figs 1C and F and S1B) and because RAD21 is the closing subunit of the cohesin ring complex with functions in chromatin organization, DNA repair and sister chromatid cohesion (Uhlmann, 2025). As a general hallmark, direct substrates of p97 are trapped by the ATPase-deficient p97-E578Q mutant that binds but cannot release substrates (Hulsmann et al, 2018). Consistent with that, co-immunoprecipitation revealed that RAD21 interacted with p97-E578Q whereas the interaction with p97 wild-type was not detectable, indicating Rad21 is a substrate of p97 (Fig 1G). Together, these data show that RAD21 is a direct target protein of the p97^Ufd1-Npl4^ complex.

We next asked whether p97^Ufd1-Npl4^ may assist degradation of RAD21. Intriguingly, whereas p97 inhibition by CB-5083 led to accumulation of ubiquitylated RAD21, such accumulation was not detected upon treatment with the proteasome inhibitor MG-132 (Fig 1H), indicating that ubiquitylated RAD21 was not targeted to the proteasome downstream of p97 but rather recycled. Consistent with that, cycloheximide chase experiments revealed that RAD21 was stable over the respective period of time of 8 h, and rates were not affected by p97 inhibition in contrast to an established p97 substrate, PPP1R15B (Hulsmann et al, 2018; Fig S1C). As expected, accumulation of RAD21 ubiquitylation upon p97 inhibition was blunted by pharmacological inactivation of E1 ubiquitin-activating enzyme with MLN7243 (Fig S1D). In contrast, inhibition of neddylation with the Nedd8 E1 inhibitor MLN4924 led to a partial reduction of ubiquitylation, pointing to an involvement of a cullin-RING E3 ubiquitin ligase in addition to yet unidentified E3 ligases (Fig S1D). In contrast, blocking SUMOylation by TAK981 only mildly affected ubiquitylation (Fig S1D). Based on these data, we conclude that Rad21 is regulated by ubiquitylation involving p97-mediated recycling.

### p97^Ufd1-Npl4^ regulates chromatin association of RAD21

**Figure 2.**
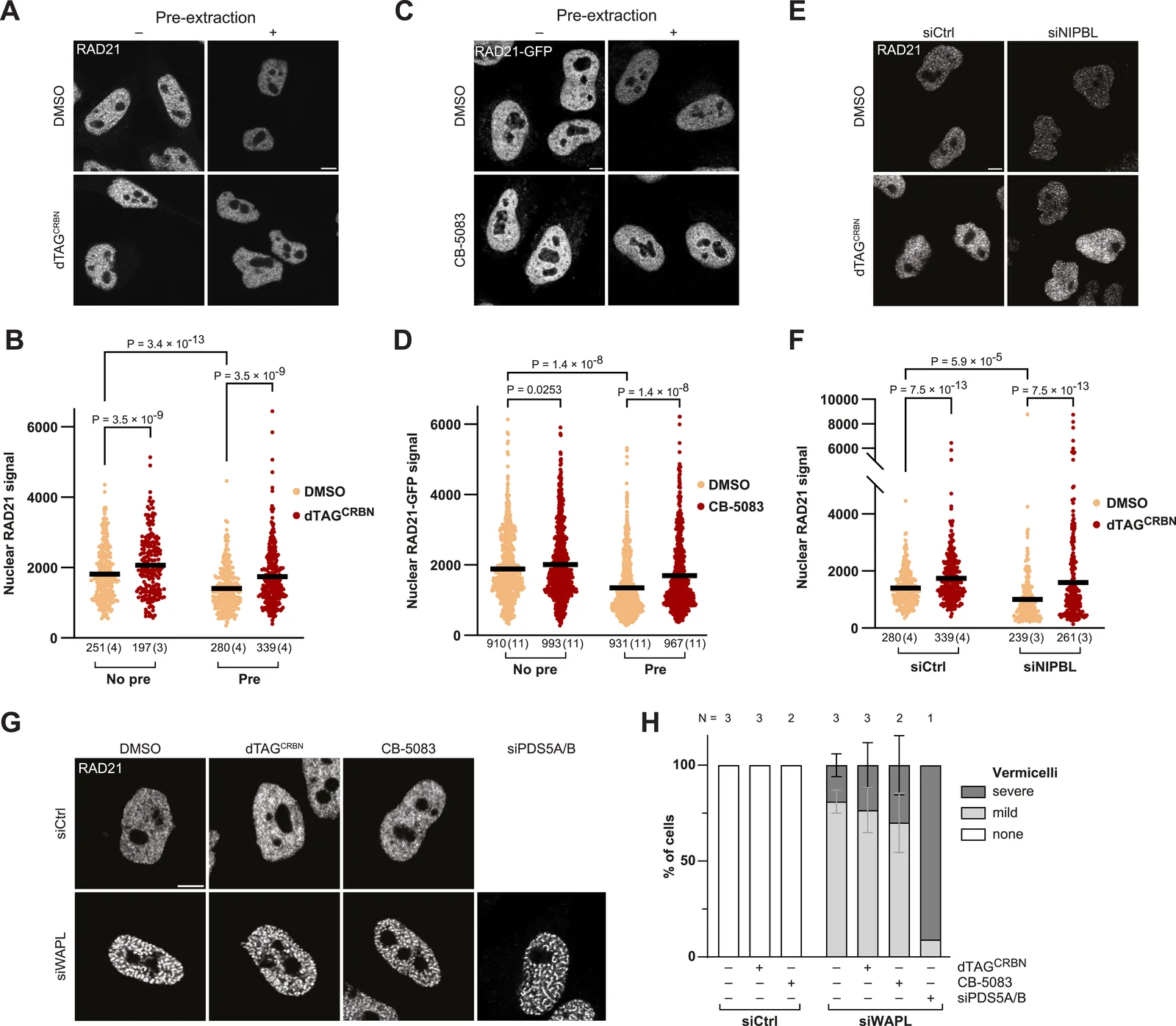
p97^Ufd1-Npl4^ regulates levels of RAD21 on chromatin. **(A)** Confocal immunofluorescence microscopy of endogenous RAD21 in HeLa Ufd1^FKBP12^ cells treated with dTAG (1 µM) to degrade Ufd1 or vehicle alone for 5 h. Cells were either fixed directly or after brief pre-extraction in buffer containing 0.1% Triton X-100 to specifically visualize chromatin-bound RAD21. **(B)** Quantification of (A). Analyzed number of cells (and biological replicates in parenthesis) are indicated. **(C)** Experiments as in (A) but in HeLa cells stably expressing RAD21-GFP and treated with the p97 inhibitor CB-5083 (5 µM) or vehicle alone for 4 h. **(D)** Quantification (of C). Analyzed number of cells (and biological replicates in parenthesis) are indicated. **(E)** Experiments as in (A) in Ufd1^FKBP12^ cells combining dTAG treatment with siRNA-mediated depletion of the cohesin-loader NIPBL (siNIPBL) as indicated. Note that siNIPBL reduces chromatin-bound RAD21, as expected, which is partially rescued by Ufd1 elimination. **(F)** Quantification of (E). Analyzed number of cells (and biological replicates in parentheses) are indicated. Note that siCtrl values from Fig 2B are shown for comparison. **(G)** RAD21 immunofluorescence after indicated treatment combinations of dTAG-induced Ufd1 degradation (1 µM, 4 h), p97 inhibition (CB-5083; 5 µM, 4 h), or PDS5A plus PDS5B siRNA with siRNA-mediated depletion of the cohesin unloader WAPL (siWAPL; 72 h). Note hyper-condensation of RAD21 specifically by siWAPL that is further increased by PDS5A/B depletion but not affected by inactivation of p97^Ufd1-Npl4^. **(H)** Quantification of (G). Number of biological replicates indicated above. **(B, D, F)** Significance determined by two-way ANOVA (one-way ANOVA for D) with Tukey’s multiple comparison test. Bars indicate means. **(A, C, F, G)** Scale bar, 5 µm.

**Figure S2.**
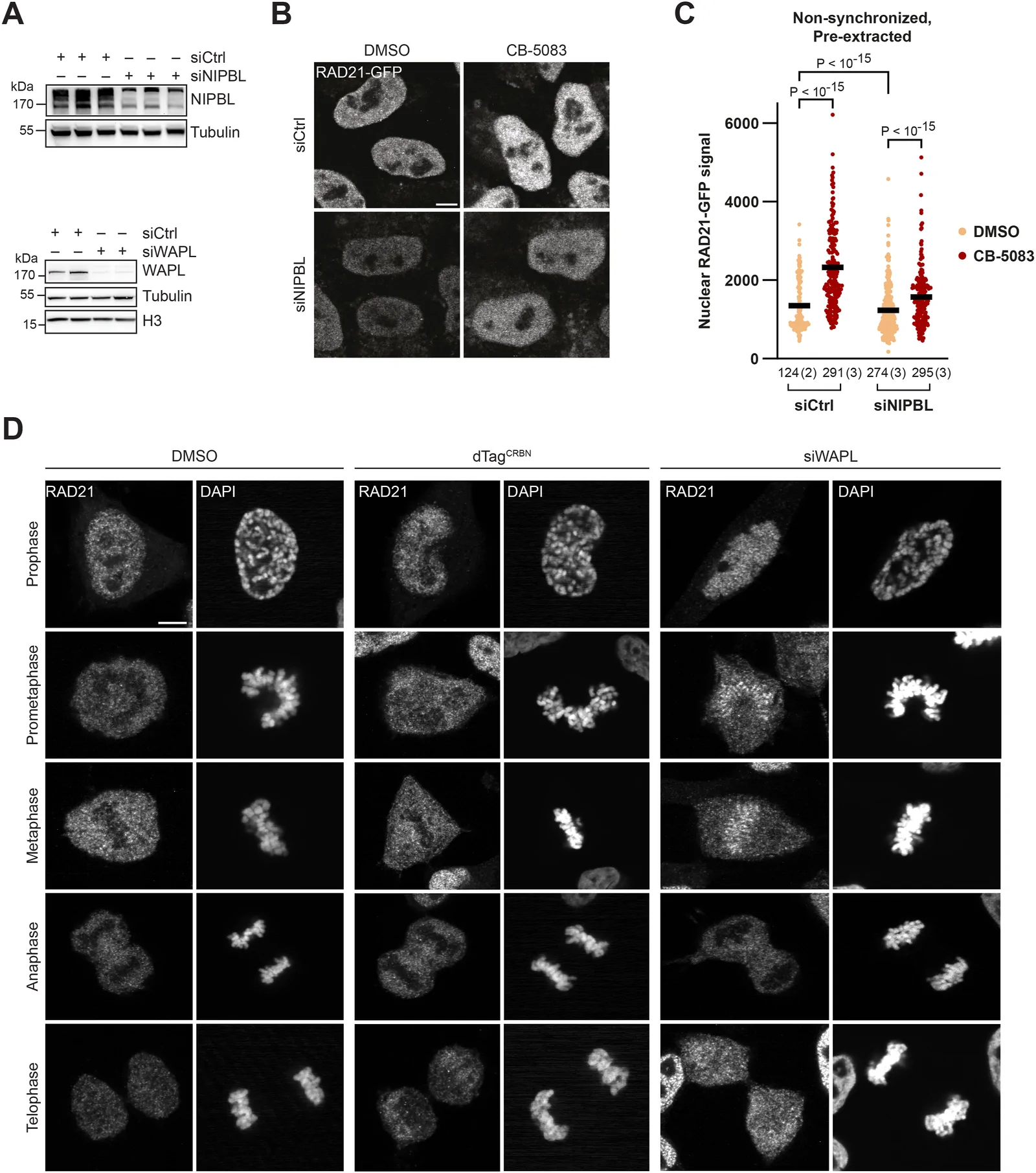
Related to Fig 2. **(A)** Western blot verification of siRNA-mediated depletion of WAPL (72 h) or NIPBL (72 h), as indicated. Repeats represent technical replicates with the identical siRNAs. **(B)** Stable HeLa RAD21-GFP cells were transfected with siRNA targeting the cohesin loader NIPBL (siNIPBL; 72 h) or with a scrambled control (siCtrl; 72 h). Cells were then treated with the p97 inhibitor CB-5083 (5 µM) or vehicle alone for 4 h. Scale bar, 5 μm. **(C)** Quantification of (B). Total number of cells indicated under each condition with biological replicates in parentheses. Significance determined by two-way ANOVA with Tukey’s multiple comparison test. Bars indicate means. **(D)** Mitotic cells immunostained for endogenous RAD21 under conditions of WAPL depletion (siWAPL, 72 h) or Ufd1 degradation (dTAG, 8 h). DAPI staining was used to identify mitotic cells based on chromatin morphology. Note that RAD21 is retained on chromosomes during prometaphase and metaphase in WAPL but not in Ufd1-depleted cells. Representative images across two independent biological replicates. Scale bar, 5 μm.

p97 can regulate protein localization independently of the proteasome by extracting target proteins from structures such as chromatin (van den Boom & Meyer, 2018). We therefore asked whether the p97^Ufd1-Npl4^ complex may extract RAD21 from chromatin. Nuclear localization of RAD21 was increased upon Ufd1 degradation or p97 inhibition as observed by confocal fluorescence microscopy (Fig 2A–D). Of note, mild pre-extraction of the soluble pool of RAD21 using a Triton-X100 buffer before fixation revealed a significant increase of a stably chromatin-associated RAD21 pool both upon Ufd1 degradation or upon p97 inhibition with CB-5083 (Fig 2A–D). RAD21 is loaded as part of the cohesin ring onto DNA as mediated by the protein NIPBL (Nasmyth & Haering, 2009). Depletion of NIPBL by siRNA reduced the amount of pre-extraction-resistant RAD21, as expected (Figs 2E and F and S2A). Notably, in NIPBL-depleted cells in which we induced degradation of Ufd1 (Fig 2E and F) or inhibited p97 (Fig S2B and C), RAD21 levels were partially restored, suggesting that NIPBL-mediated loading of RAD21 and its p97^Ufd1-Npl4^-mediated extraction are antagonizing processes that regulate RAD21 levels on chromatin.

The established pathway for discharge of cohesin from chromatin is governed by the cohesin unloading protein WAPL (Tedeschi et al, 2013). We confirmed that depletion of WAPL led to an accumulation of RAD21 on chromatin resulting in hyper-condensed chromosomes termed “vermicelli” (Figs 2G and H and S2A). Acute degradation of Ufd1 or inhibition of p97 did not induce vermicelli and the combination of WAPL depletion and Ufd1 degradation did not further increase the vermicelli, as opposed to depletion of PDS5A and PDS5B that increased the effect of WAPL depletion (Fig 2G and H). Moreover, in contrast to WAPL depletion, lack of Ufd1 had no apparent effect on cohesin removal from chromosome arms in mitosis (Fig S2D). This suggests that WAPL and p97-mediated regulation of cohesin are independent processes and that the p97 complex may only act under certain conditions and on a subpopulation of cohesin.

### p97^Ufd1-Npl4^ extracts cohesin during S phase

**Figure 3.**
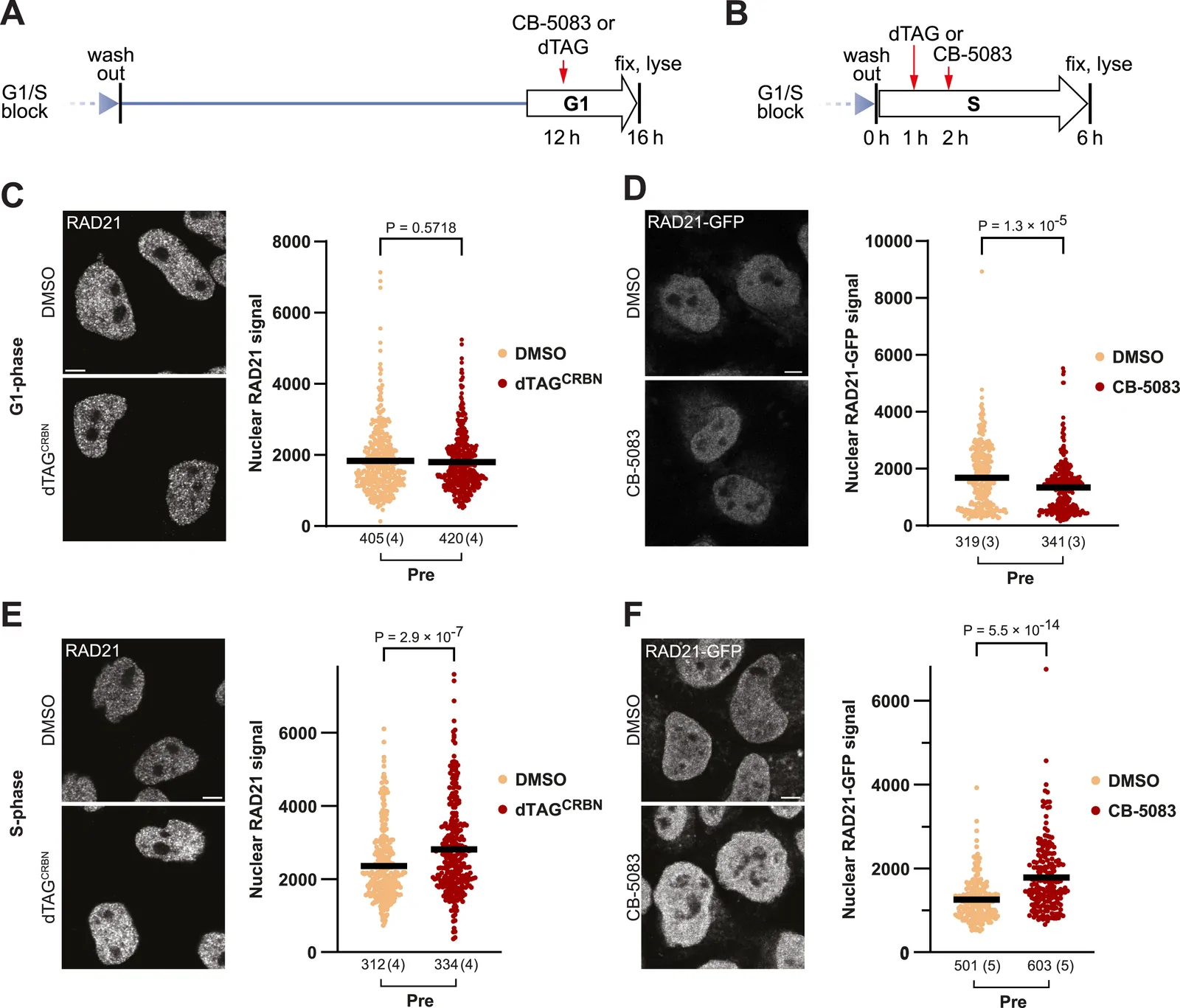
p97^Ufd1-Npl4^ extracts cohesin from chromatin during S phase. **(A, B)** Cell cycle synchronization and treatment schemes after a double thymidine block at G1/S and release for 16 h (G1 phase) or up to 6 h (S phase). dTAG or CB-5083 treatments at indicated time points. See Fig S3A and B for synchronization validation. **(C)** HeLa Ufd1^FKBP12^ cells synchronized in G1 and treated as indicated in (A) with dTAG (1 µM, 4 h) were preextracted and immunostained for endogenous RAD21. **(D)** HeLa cells stably expressing RAD21-GFP were synchronized and treated in G1 as indicated in (A) with CB-5083 (5 µM, 4 h) followed by preextraction, fixation and imaging (E) HeLa Ufd1^FKBP12^ cells synchronized in S phase and treated as indicated in (B) with dTAG (1 µM, 5 h) were preextracted and immunostained for endogenous RAD21. **(F)** HeLa cells stably expressing RAD21-GFP were synchronized in S-phase and treated as indicated in (B) with CB-5083 (5 µM, 4 h) followed by preextraction and imaging. **(C, D, E, F)** Total number of cells indicated under each condition with biological replicates in parentheses. Significance determined by two-tailed unpaired *t* test. Bars indicate means. Scale bar, 5 µm.

**Figure S3.**
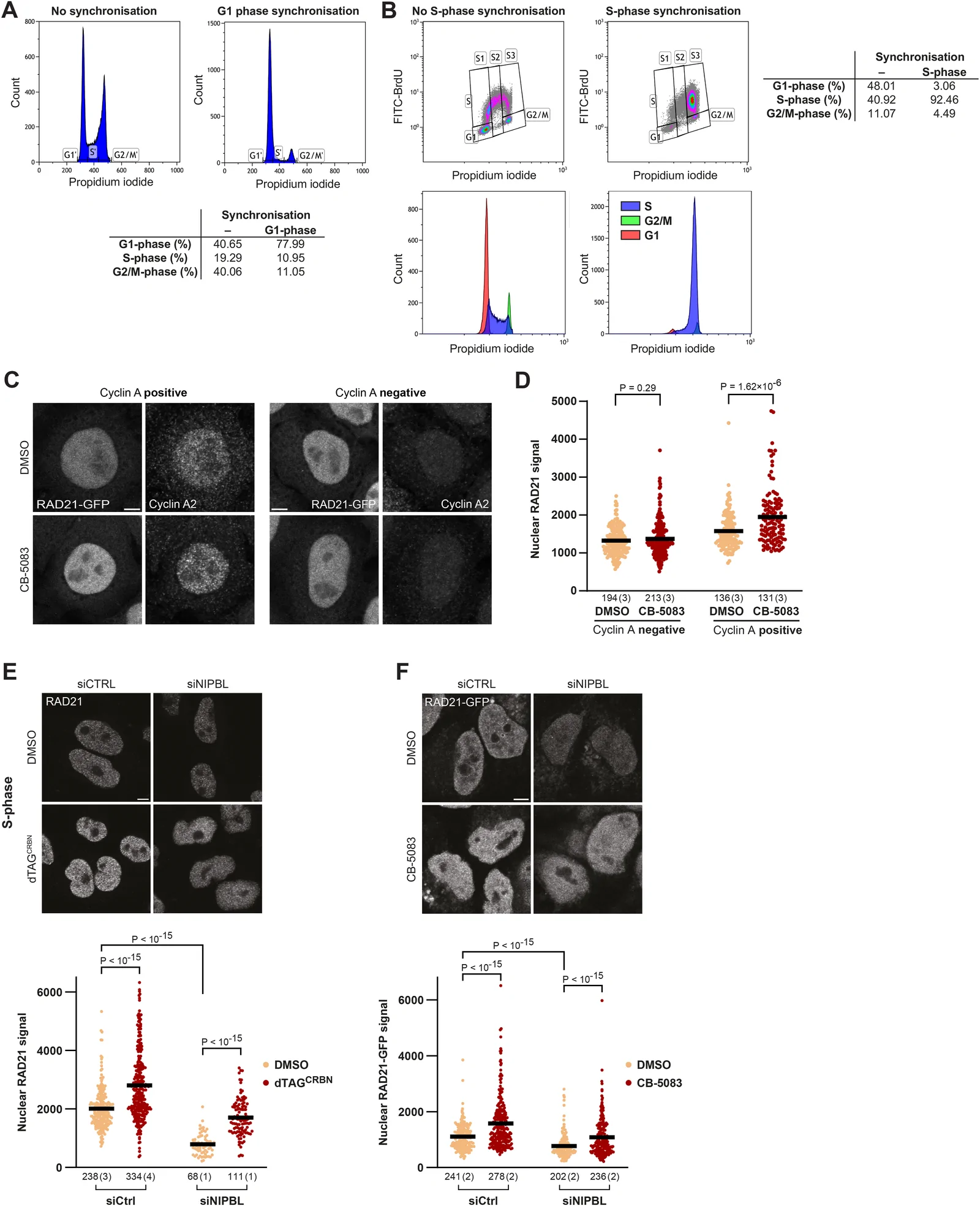
Related to Fig 3. **(A)** Validation of G1 phase synchronization (16 h release from double-thymidine block) using flow cytometry after propidium iodide staining. The two plots represent data from one of two independent synchronizations. **(B)** Validation of S phase synchronization (6 h release from double-thymidine block) using flow cytometry after BrdU incorporation, and BrdU and propidium iodide staining. Note that cells accumulate in late S-phase (S3) as expected. Representative plots from 2 independent synchronizations. S1, early S-phase; S2, mid-S-phase; S3, late S-phase. **(C)** Categorization of unsynchronized stable HeLa RAD21-GFP cells according to expression of cyclin A2 detected by immuno-staining after treatment with or without CB-5083. **(D)** Quantification of (C). Note that Cyclin A2-positive (S/G2) cells showed a significant increase of nuclear RAD21-GFP upon CB-5083 treatment, but not cyclin A2-negative (G1) cells. **(E)** Rescue of RAD21 on chromatin after NIPBL depletion by dTAG-induced Ufd1 degradation in S-phase. HeLa Ufd1^FKBP12^ cells were depleted of NIPBL (72 h), synchronized in S-phase, treated with dTAG (1 µM, 5 h) according to scheme in Fig 3B, and immunostained for RAD21 after preextraction. **(F)** Rescue of RAD21 on chromatin in S-phase after NIPBL depletion by p97 inhibition. Stable RAD21-GFP Hela cells were depleted of NIPBL (72 h), synchronized in S-phase, treated with CB-5083 (5 µM, 4 h) according to scheme in Fig 3B, and immunostained for RAD21 after preextraction. **(D, E, F)** Total number of cells indicated under each condition with biological replicates in parentheses. Significance determined by two-way ANOVA with Tukey’s multiple comparison test. Bars indicate means.

At least two pools of cohesin exist on chromatin (Uhlmann, 2025). One is the dynamically associated pool that mediates DNA loop extrusion and, thus, organization of the chromatin, which is dominant in the G1 phase of the cell cycle. In contrast, in S-phase, cohesin needs to be removed for replication fork progression and cohesive cohesin has to be loaded that locks the sister chromatids together throughout G2 phase until mitosis. Using a double thymidine block and a final release from the G1/S block for up to 6 or 16 h, we synchronized cells for treatment and analysis specifically in S and G1 phases, respectively (Figs 3A and B, and S3A and B), and analyzed RAD21-GFP in stable cells by microscopy. Of note, when cells were treated and analyzed while in G1, we did not observe a significant increase in chromatin-associated RAD21-GFP upon p97 inhibition (Fig 3C and D). In contrast, when cells were treated with either p97 inhibitor or Ufd1 degrader while in S phase, a significant increase of chromatin-associated RAD21 was apparent (Fig 3E and F) indicating that p97 and Ufd1 regulate cohesin specifically in the S phase. As an alternative approach, we categorized unsynchronized cells according to expression of the cell cycle marker cyclin A2 as detected by immuno-staining. In line with the synchronization experiments, chromatin-associated RAD21 was increased upon p97 inhibition in cyclin-A2-positive cells, which are in S or G2 phase, but not in cyclin A2-negative cells that are in G1 phase (Fig S3C and D). In the S phase-synchronized cells, Ufd1 degradation or p97 inhibition again partially rescued the loss of RAD21 upon NIPBL depletion (Fig S3E and F). These data show that p97^Ufd1-Npl4^ removes part of cohesin from DNA specifically during S phase.

### The p97^Ufd1-Npl4^ complex targets cohesin in S-phase to safeguard against replication-associated DNA damage

**Figure 4.**
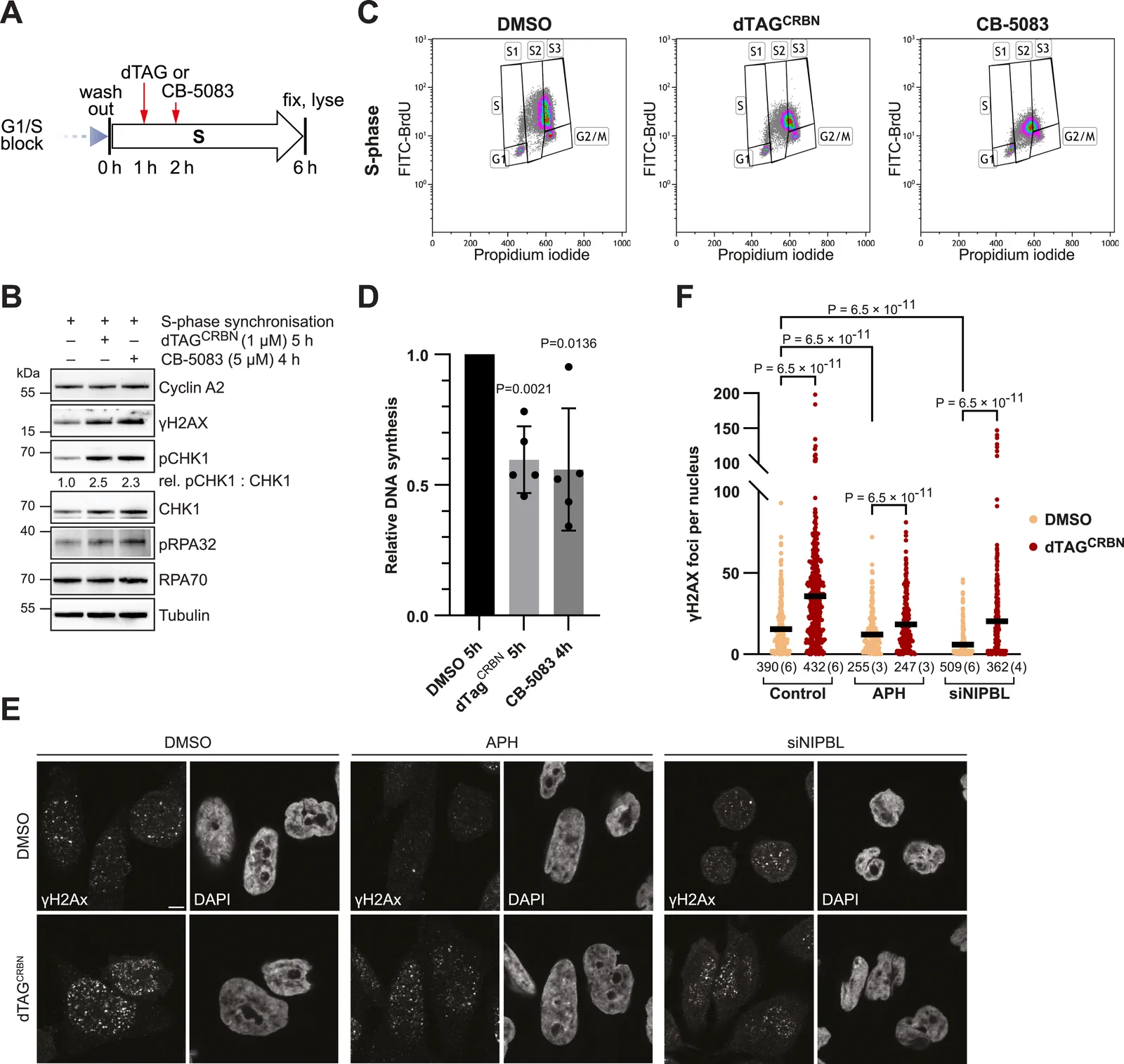
The p97^Ufd1-Npl4^ complex targets cohesin in S-phase to safeguard against replication-associated DNA damage. **(A, B)** Inactivation of p97^Ufd1-Npl4^ in S-phase leads to DNA damage checkpoint activation. HeLa Ufd1^FKBP12^ cells were synchronized and treated in S-phase according to scheme in (A), lysed and subjected to Western blot analysis with indicated antibodies (B). Relative pCHK1 to CHK1 band intensities are indicated. **(C)** Inactivation of p97^Ufd1-Npl4^ impedes DNA synthesis. Cells were synchronized in S-phase and treated with dTAG (1 µM, 5 h) or CB-5083 (5 µM, 4 h) according to the scheme in (A). BrdU incorporation was initiated 30 min before fixation and analyzed by flow cytometry. **(D)** Quantification of BrdU incorporation in (C) as a measure for DNA synthesis. n = 5 biological replicates. Means ± S.D. Significance determined by one-sample *t* test against a hypothetical value of 1. **(E)** HeLa cells were synchronized in S-phase and treated according to (A). Cells were either treated with dTAG (1 µM, 5 h) to eliminate Ufd1 in combinations with the replication inhibitor aphidicolin (APH; after double-thymidine washout, 1 µM for 6 h) or NIPBL siRNA (72 h) or vehicle alone (DMSO) as indicated. Cells were fixed and immunostained for γH2AX along with DAPI. **(F)** Quantification of (E). Total number of cells indicated under each condition with biological replicates in parentheses. Significance determined by two-way ANOVA with Tukey’s multiple comparison test. Bars indicate means.

**Figure S4.**
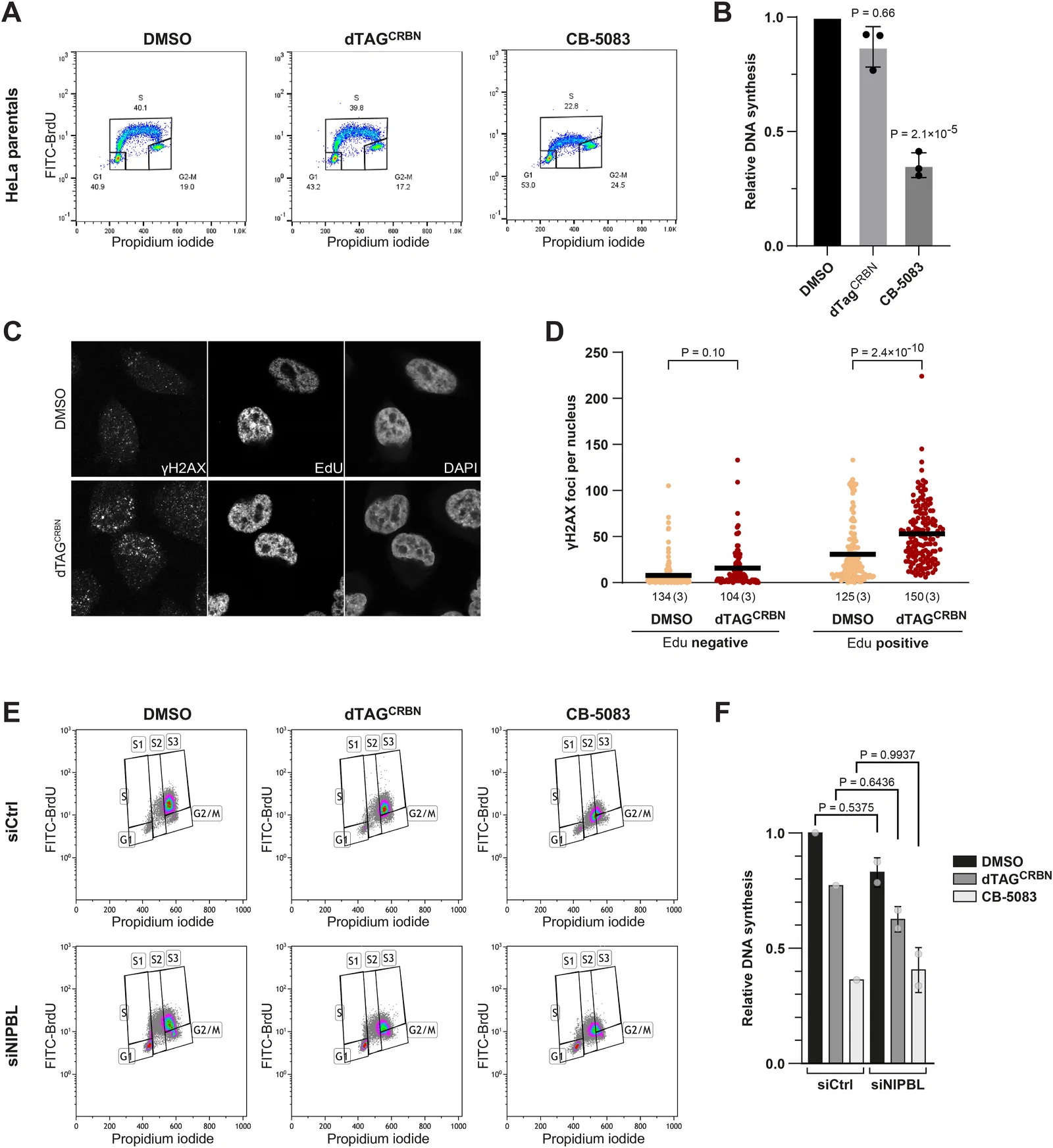
Related to Fig 4. **(A)** Effect of dTAG^CRBN^ or CB-5083 treatments on DNA synthesis in HeLa parental cells lacking the FKBP12 tag on Ufd1. Flow cytometry detecting BrdU incorporation and propidium iodide staining of indicated cell populations. **(B)** Quantification of (A). n = 3 biological replicates. Means ± S.D. Significances determined by one-sample *t* tests against a hypothetical value of 1. **(C)** Categorization of unsynchronized Ufd1^FKBP12^ cells according to EdU incorporation and detection of γH2AX foci using immuno-staining. **(D)** Quantification of (C). Total number of analyzed cells indicated (n = 3 biological replicates). Note the specific increase of γH2AX foci in EdU-positive (S phase) cells treated with dTAG^CRBN^. Significance determined by two-way ANOVA with Tukey’s multiple comparison test. Bars indicate means. **(E)** DNA replication assessment after BrdU incorporation using flow cytometry in conditions of NIPBL depletion (72 h) in combination with vehicle alone, Ufd1 degradation (dTAG; 1 µM, 5 h) or p97 inhibition (CB-5083; 5 µM, 4 h) as indicated. HeLa Ufd1^FKBP12^ cells were synchronized in S-phase and treated according to Fig 4A. BrdU incorporation occurred during the 30 min before fixation. **(F)** Quantification of (A). BrdU fluorescence intensity was used as a readout of relative DNA synthesis. The mean BrdU fluorescence measured in G1 and G2/M phases was averaged and subtracted from S-phase BrdU intensity to remove background signal. The resulting BrdU fluorescence values were normalized to the DMSO vehicle control. n = 1 biological repeat with 1 technical repeat of controls and 2 technical repeats of siNIPBL. Significance determined by one-way ANOVA with Tukey’s multiple comparison test. Bars indicate means ± S.D.

We next aimed to explore the significance of p97^Ufd1-Npl4^-mediated extraction of cohesin during S-phase. In the S phase-synchronized cells, we noted that the DNA damage checkpoint was activated upon Ufd1 degradation or p97 inhibition with phosphorylation of CHK1, RPA32 and H2AX (Fig 4A and B). Moreover, under these conditions, BrdU incorporation was reduced compared with untreated Ufd1^FKBP12^ cells according to flow cytometry indicating compromised DNA replication upon inactivation of p97^Ufd1-Npl4^ (Fig 4C and D). Of note, treatment with the dTAG^CRBN^ compound of parental cells harboring wild type Ufd1 without the FKBP^F36V^ tag did not result in a significant inhibition of BrdU incorporation indicating that the effect on DNA replication is specific to elimination of Ufd1 and not to the compound itself (Fig S4A and B). Previous work showed a role of p97/CDC-48 in replication licensing through degradation of CDT1 (Franz et al, 2011; Raman et al, 2011). However, in our setup, replication was affected even in cells in which we induced degradation of Ufd1 in synchronized cells 1 h after release from the G1/S block when replication licensing is already completed. Importantly, under these conditions, we observed a significant increase of γH2AX foci in cells fixed 6 h hours after the G1/S release in cell lacking Ufd1 compared with control (Fig 4E and F). In parallel, we scored unsynchronized cells by incorporation and detection of the nucleotide analog 5-ethynyl-2′-deoxyuridine (EdU) to identify S-phase cells. In line with the synchronization experiments, EdU-positive (S-phase) cells showed a larger increase in γH2AX upon Ufd1 degradation than EdU-negative cells (Fig S4C and D). The increase in γH2AX foci in S-phase synchronized cells was largely reduced by concomitant inhibition of replication with aphidicolin indicating that the lack of p97^Ufd1-Npl4^ function during S phase caused replication-associated DNA damage (Fig 4E and F). Replication-associated DNA damage induced by the degradation of Ufd1 was linked to cohesin dynamics because concomitant siRNA-mediated depletion of the cohesin loader NIPBL reduced the number of γH2AX foci caused by Ufd1 elimination during S phase (Fig 4E and F). Of note, this effect was not indirect through slowing down replication, because NIPBL depletion did not largely reduce replication (Fig S4E and F). These data demonstrate that an important role of p97^Ufd1-Npl4^ during S phase is to remove part of cohesin from DNA to prevent replication-associated DNA damage.

## Discussion

VCP/p97^Ufd1-Npl4^ has drawn attention as a critical regulator of ubiquitin-mediated processes on chromatin. By combining a rapid induced-degradation approach specific to the Ufd1 subunit that inactivates the prominent ubiquitin adapter for p97, in this study, we identified a set of ubiquitin modified target proteins that sheds light on the functions of p97 in chromatin-associated processes. By focusing on the closing subunit of the cohesin ring, RAD21, as a candidate, we reveal a critical role of p97 in extracting cohesin from DNA that is different from previously known cohesin unloading mechanisms.

WAPL is the major unloader for cohesin (Uhlmann, 2025). Compromised WAPL function leads to hyper-condensed DNA with excessive RAD21 on chromatin because cohesin is loaded but not unloaded (Tedeschi et al, 2013). This is relevant in non-cycling or G1 cells, as dynamic association and dissociation of cohesin leads to DNA loop extrusion and governs organization of the chromatin. We do not observe changes in cohesin association with chromatin in G1 upon p97^Ufd1-Npl4^ inactivation, nor do we detect persistence of cohesion on chromosome arms during mitosis, which is also removed by WAPL in preparation for chromosome segregation. Instead, we find that p97^Ufd1-Npl4^ extracts cohesin specifically in S-phase, suggesting that this activity is linked to replication. In line with that notion, we see an impairment of replication upon acute Ufd1 elimination. Importantly, failure to remove part of cohesin during S phase after elimination of Ufd1 results in replication-associated DNA damage.

In principle, the observed damage could in part result from unrepaired spontaneous damage during S phase, because p97 is involved in DNA damage repair in many ways (Vaz et al, 2013). However, we observed that the emergence of damage is mitigated by reducing cohesin loading in cells depleted of NIPBL. We therefore speculate that a subpopulation of DNA-entangled cohesion needs to be removed by p97^Ufd1-Npl4^ to allow replication fork progression. In this model, if p97 fails, the replication fork would stall due to persisting cohesin followed by a possible collapse that leads to the observed replication-associated DNA damage in the absence of Ufd1. This is consistent with the previous observation that the p97 complex ensures replication fork progression as shown also in DNA combing fiber assays (Franz et al, 2016b). The cohesin-directed pathway may have developed to act as a safe-guard mechanism if the canonical cohesin unloading by WAPL fails for example due to damage to its components. It is tempting to speculate that collision of the replication fork with persistent cohesin rings may be the trigger to induce RAD21 ubiquitylation in order to attract the p97 complex for unloading. In yeast, cohesin subunits are ubiquitylated by Rsp5 for unloading by Cdc48 during replication (Frattini et al, 2017) consistent with our model for human cells. The partial effect of a neddylation inhibitor on RAD21 inhibition found here may implicate a Cullin-RING ubiquitin ligase in human cells. A recent report finds that replications forks can push cohesin rings along the DNA double strand and even convert them to cohesive cohesin at replication termination (Cameron et al, 2024). However, it remains conceivable that a fraction of cohesin must still be removed during replication. Of note, we do not find evidence for RAD21 degradation downstream of p97-mediated extraction from chromatin. It would therefore be interesting to identify the deubiquitinating enzyme that removes ubiquitin downstream of p97 and possibly recycles RAD21 for re-use in sister chromatid cohesion during replication.

## Materials and Methods

### Plasmids

Rad21-mEmerald (plasmid # 54248), pEGFP-STAG2 (plasmid # 31972), pCAIP-hXRCC1-WT-eGFP (plasmid # 206032) and pcDNA5-FRT-TO-FLAG-MORF4L1 (plasmid # 86874) were ordered from Addgene. HUS1 and MCM7 were cloned into pEGFP-C1 (Clontech) and pEGFP-N1 (Clontech), respectively. GFP empty vectors were PCR-amplified to generate overlaps compatible with Gibson assembly. To generate inserts, human HUS1 and human MCM7 were amplified from HeLa cDNA (primers TTC​GAA​TTC​TGC​AGT​CGA​CGA​TGA​AGT​TTC​GGG​CCA​AG and GGA​TCC​CGG​GCC​CGC​GGT​ACC​TAG​GAC​AGC​GCA​GGG​ATG for HUS1; ATC​TCG​AGC​TCA​AGC​TTC​GAA​TGG​TGG​TGG​CCA​CTT​AC and GGT​ACC​GTC​GAC​TGC​AGA​ATG​ACA​AAA​GTG​ATC​CGT​GTC for MCM7) which generated complementary overlaps. Following Gibson assembly, the resulting plasmids were referred to as GFP-HUS1 and MCM7-GFP. The HA-Ubiquitin, pcDNA5-p97-myc and pcDNA5-p97-E578Q-myc plasmids were previously described (Ritz et al, 2011; Hulsmann et al, 2018). pRRL-PGK-DHB-mKate2 was generously gifted by the Peters lab, IMP Vienna.

### Cell lines

A HeLa Ufd1-dTAG stable cell line was generated by introducing a transgene of Ufd1 containing a C-terminal HA-FKBP12(F36V) tag into HeLa FRT cells via the Flp-In system, followed by a knockout of the UFD1 gene using CRISPR Cas9 technology (KO plasmid: sc-403304; Santa Cruz; HDR plasmid: sc-403304-HDR; Santa Cruz). Inducible HEK293 stable cell lines which express p97-WT-myc-strep or p97-EQ-myc-strep were previously generated via the Flp-In T-Rex system (Invitrogen) according to the manufacturer’s protocol (Hulsmann et al, 2018). Hela Rad21-GFP stable cells were kindly provided as a gift by the Peters lab, IMP Vienna.

### Mammalian cell culture

Stable HeLa Rad21-GFP cells were cultured in DMEM containing 10% FBS and 1% Penicillin/Streptomycin. HEK293 p97-WT and p97-EQ cells were cultured in medium as described above, supplemented with 15 μg/ml blasticidin S and 100 μg/ml hygromycin B. Expression was induced with 1 μg/ml doxycycline for 24 h. HeLa Ufd1^FKBP12^ cells were cultured in DMEM medium, as described above, supplemented with 15 μg/ml blasticidin S, 100 μg/ml hygromycin B and 0.5 µg/ml puromycin. Cells were maintained in incubators under standard growth conditions, 37°C and 5% CO2. Cells were transfected with plasmids using Lipofectamine 2000 (Thermo Fisher Scientific) or with 10 nM siRNA using RNAiMAx (Thermo Fisher Scientific) according to the manufacturer’s instructions. Transfected cells were analyzed after 24 h in the case of plasmids and 48 or 72 h in the case of siRNA.

### RNA interference

Previously established siRNAs targeting WAPL and NIPBL were purchased from Microsynth:

siWAPL #1 5′-CGGACUACCCUUAGCACAAdTdT-3′ (Kueng et al, 2006), siNIPBL #1 5′-GCAUCGGUAUCAAGUCCCAdTdT-3′ (Watrin et al, 2006; Zhou et al, 2017), siPDS5A 5′-GCUCCAUAUACUUCCCAUGdTdT-3′ (Wutz et al, 2017), siPDS5B 5′-GAGACGACUCUGAUCUUGUdTdT-3′ (Wutz et al, 2017).

### Cell treatments

To induce DNA damage, cells were subjected to ionizing irradiation (IR, 2 Gy) (Xstrahl Sys CIX2). For p97 inhibition, cells were treated with 5 μM of CB-5083 (#S8101; Selleckchem) for the periods indicated. To induce Ufd1^FKBP12^ degradation, cells were treated with 1 µM SLF’ Thalidomide (dTAG-7; Hölzel) over the time periods indicated. To inhibit protein synthesis, cells were treated with 50 µg/ml cycloheximide for the times indicated. To inhibit the proteosome, cells were treated with 20 µM of MG132 (Merck) for the times indicated. To inhibit E1 ubiquitin, Nedd8 and SUMO-activating enzymes, cells were treated with 50 μM MLN7243 (Hycultec), 1 μM MLN4924 (Hycultec) or 0.5 μM TAK981 (MedChemExpress), respectively, for the times indicated. To monitor autophagy, V-ATPase was inhibited using 100 nM of Bafilomycin A1 (Biomol). To block DNA replication, cells were treated with 1 µM of the DNA polymerase inhibitor aphidicholin (Hölzel).

### Cell synchronization

HeLa Rad21-GFP cells and HeLa Ufd1^FKBP12^ cells were synchronized using a double thymidine block and release approach. Briefly, cells were treated with 2 mM thymidine for 16 h, released for 8 h and subjected to 2 mM thymidine a second time for 16 or 24 h. To obtain G1 cells, cells were released for 16 h after the double thymidine block. To obtain cells in late S-phase, cells were released for 6 h after the double thymidine block. Cells were treated with dTAG-7 or CB-5083 for the last 4–5 h of the release, refer to individual experiments.

### Flow cytometry for cell cycle analysis

Cells were labeled with 10 µM BrdU (Sigma-Aldrich) within the last 30 min of treatment. Cells were harvested by trypsinization, washed in PBS and fixed in −20°C MeOH overnight. Following fixation, cells were washed with PBS/0.01% Triton and incubated for 30 min in 2 M HCl/0.5% Triton. After three PBS/0.01% Triton wash steps, cells were stained with a 1/5 dilution of anti-BrdU-FITC (BD Biosciences) in PBS/1% BSA/0.01% Triton for 1 h in the dark. After an additional wash step, cells were incubated with 25 µg/ml propidium iodide prepared with 10 µg/ml RNaseA (Macherey-Nagel) for 1 h at 37°C. Centrifugation was performed at 850*g* for 1 min. On the MACSQuant MQ16 flow cytometer, 50,000 events/sample were acquired with a low flow rate and gentle mixing. The proportions of PI-positive cells and BrdU-incorporating cells were determined with Kaluza Analysis software.

### Fluorescence confocal microscopy

Cells were cultured on Poly-L-lysine or Poly-D-lysine coated coverslips (Neuvitro) and fixed in 4% paraformaldehyde (PFA) at room temperature. To remove soluble proteins, prior to PFA fixation, cells were pre-extracted with 0.1% Triton X-100 in PBS for 3 min on ice. All samples were then permeabilized with 0.1% Triton X-100 in PBS and blocked in PBS with 3% BSA + 0.1% Triton X-100 +0.1% saponin. Following immunofluorescence staining, cells were mounted in ProLong Gold (Thermo Fisher Scientific).

Fluorescence confocal microscopy was performed on a Leica TCS SP8X Falcon confocal microscope (Leica Microsystems), equipped with HyDs SMD detectors, HC PL APO 63x/1.4 Oil CS2 objective and the Leica Application Suite X (LAS-X) software version 3.5.7. Fluorophores were excited by a white-light laser, Argon laser, and/or a 405-diode laser. Images were acquired at 1.5× zoom in a 1024 × 1024 x format with a bit depth of 12 bit.

When indicated, cells were labeled with the thymidine analog 5-ethynyl-2′ deoxyuridine (EdU, 5 µM) prior to fixation. EdU was added after 1 h of dTAG treatment and incubated for 1 h before being washed out. EdU incorporation was detected using the EdU Click-647 ROTI kit for imaging (Carl Roth) according to the manufacturer’s instructions, enabling visualization by fluorescence microscopy using 633 nm excitation.

### Image analysis

Images were processed using ImageJ software (https://imagej.net/Fiji). Automated quantifications were performed using Cell Profiler software version 4.2.8 (Carpenter et al, 2006). Generation of graphs and statistical analysis were performed using Excel 2016 (Microsoft Corporation) and GraphPad Prism 10.3 (GraphPad Software).

### Western blot analysis and immunoprecipitation

Cells were lysed 24 h after transient transfection and after indicated treatments. To ensure the extraction of nuclear proteins, a 1% SDS buffer was used when indicated (1% SDS, 10 mM Tris, 1 mM EDTA, pH 8, Roche Complete EDTA-free protease inhibitors, Roche PhosStop easy pack phosphatase inhibitors, 1 mg/ml N-ethylmaleimide). In cases where only membrane permeabilization was required, a 1% Triton X-100 buffer was used as indicated (1% Triton X-100, 150 mM KCl, 5 mM MgCl2, 50 mM Tris–HCl pH 7.4, 5% glycerol, 2 mM β-mercaptoethanol, Roche Complete EDTA-free protease inhibitors, Roche PhosStop easy pack phosphatase inhibitors, 1 mg/ml NEM). Cells were incubated with lysis buffer for 10 min before lysates were collected via centrifugation (15 min, 16,000*g*, 4°C). In the case of the SDS-lysates, DNA was sheared either via benzonase (25 U/ml) or using a diagenode bioruptor plus, 30 s ON, 30 s OFF for 20–30 cycles. Protein concentration was determined via BCA assay and samples were loaded onto SDS–PAGE gels accordingly. Resolved proteins were transferred to nitrocellulose membranes (Amersham, GE Healthcare) and Western blot analysis was performed with the antibodies indicated and visualized with Cytiva Amersham ECL prime detection agent or SuperSignal West Pico Chemiluminescent substrate (Pierce).

In the case of immunoprecipitation experiments, following doxycycline induction of p97-WT or p97-E578Q, lysates obtained using the 1% TritonX-100 buffer were incubated at 4°C with anti-GFP nanobodies for 2 h whilst rotating. GFP-tagged proteins were affinity-isolated using anti-GFP nanobodies.

### Denaturing immunoprecipitation

Cells were harvested with 100 µl of SDS-containing buffer (50 mM Tris pH 7.5, 1% SDS, 5 mM DTT, 1 mg/ml NEM) for 10 min on ice. Cells were scraped and SDS was diluted by addition of 900 µl dilution buffer (20 mM Tris pH 7.9, 200 mM NaCl, 0.5% NP40, Roche Complete EDTA-free protease inhibitors, Roche PhosStop easy pack phosphatase inhibitors, 1 mg/ml NEM). DNA was removed through addition of 0.5 µl Benzonase and lysates were left to rotate in the cold room for 30 min. Lysates were incubated at 4°C with anti-GFP nanobodies for 2 h whilst rotating. GFP nanobodies were washed 3 times with dilution buffer devoid of SDS and then boiled at 95°C for 5 min in 25 µl of 2x SDS sample buffer.

### Antibodies

The following primary antibodies were used: PPP1R15B/CreP (14634-1-AP; Proteintech, WB), Tubulin (mouse, T-5168; Sigma-Aldrich; WB 1:1,000), Rad21 (ab992; Abcam, WB, 1:500), Rad21 (05-908; Merck Millipore, FM, 1:400) p97 (sc-57492; Santa Cruz, WB, 1:1,000), polyubiquitin P4D1 (3936 S; cell signaling, WB, 1:1,000), HA (Clone 16B12 #MMS-101P; BioLegend, WB, 1:500), Ufd1 (sc-377265; Santa Cruz, WB), Ufd1 (611642; BD Biosciences, WB, 1:1,000), GFP (11814460001; Roche, WB, 1:500/1:1,000), Histone H3 (06-755; Merck, WB, 1:1,000) NIPBL (sc-374625; santa cruz, WB, 1:500), SMC3 (A300-060A; Thermo Fisher Scientific, WB, 1:1,000), SMC1 (A300-055A; Thermo Fisher Scientific, WB, 1:1,000), Cyclin A2 (ab16726; Abcam [6E6], Mouse, FM, WB, 1:1,000), WAPL (16875964; Proteintech, WB, 1:500), γH2Ax (#2577; cell signaling, WB, FM), γH2Ax (JBW301 05-636; Millipore, WB, FM, 1:500), γH2Ax (613401; BioLegend, WB), γH2Ax (ab11174; Abcam, WB), anti-FLAG (mouse, F3165; Sigma-Aldrich, WB, 1:500), CHK1 (sc-8408; santa cruz, WB, 1:500), pCHK1 (S345) (2348S; Cell Signaling, WB, 1:1,000), RPA70 (NB100-2204; Novus Biologicals, WB, 1:500), pRPA32 (Ser4/Ser8) (A300-245A-T; Bethyl Laboratories, WB, 1:500). HRP-coupled secondary antibodies were purchased from Bio-Rad.

### Ubiquitin profiling by mass spectrometry

HeLa Ufd1-dTAG cells were cultured for 2 wk in SILAC DMEM (Thermo Fisher Scientific) supplemented with 10% dialyzed FCS, 1% penicillin/streptomycin and either Arg0/Lys0 or Arg10/Lys8. The amino acids and dialyzed FCS were provided by the Beli lab, IMB Mainz. Once the light and heavy isotopes of arginine and lysine had been incorporated, the differentially labeled HeLa Ufd1-dTAG cells were treated with dTAG-7 or DMSO control for 8 h, or with dTAG-7 or DMSO for 4 h and irradiated 1 h after the start of treatment. Cells were washed twice with ice-cold PBS before lysis was performed on ice with a modified RIPA buffer (mRIPA) (50 mM Tris–HCl pH 7.5, 150 mM NaCl, 1 mM EDTA, 1% IGEPL CA-630, 0.1% sodium deoxycholate, protease inhibitor, phosphatase inhibitor and 1 mg/ml NEM). Following scraping, cells were left to incubate for 15 min on ice. Lysates were sonicated for 40 cycles with 30 s ON, 30 s OFF in bioruptor falcon tubes with protein extraction beads, on the Diagenode Bioruptor PLUS. The supernatant was collected following centrifugation (16, 000*g*, 15 min, 4°C). Subsequently, differentially isotope-labeled cells subjected to different treatment conditions were combined in a 1:1 ratio. The proteins were then precipitated with −20°C acetone.

Proteins were reduced by 1 mM DTT for 45 min and alkylated with 5.5 mM chloroacetamide for 30 min at room temperature. Digestion was performed overnight at room temperature with trypsin (1:100 enzyme:protein). Reactions were quenched with 0.5% TFA. Peptides were clarified by centrifugation (4,000*g*, 10 min, 4°C), desalted using Sep-Pak C18 cartridges (Waters), eluted with 50% acetonitrile, and dried by SpeedVac prior to enrichment. Desalted peptides were incubated with 80 μl diglycine-lysine (K-ε-GG) antibody resin (Cell Signaling Technology) for 4 h or overnight at 4°C with rotation (Wagner et al, 2011). Beads were washed three times with IAP buffer (50 mM MOPS/NaOH pH 7.2, 1,000 mM Na2HPO4, 50 mM NaCl) and three times with water. Enriched peptides were eluted with 0.15% TFA and separated into six fractions by micro-SCX. SCX tips were assembled using six disks from 47 mm cation exchange extraction disks were cut with a 17-gauge Hamilton syringe and placed into a 200 μl pipette tip. Micro-SCX tips were equilibrated with 50 μl of methanol, SCX elution buffer pH 4, SCX elution buffer pH 11, and 100 μl of SCX wash buffer by centrifugation at 500*g*. Acidified samples (pH < 2) were loaded on micro-SCX tips by centrifugation at 500*g* and fractionated by eluting with 100 μl of SCX elution buffers at 500*g* from lowest to highest pH (pH of SCX-buffers: 4.0/5.0/6.0/7.0/8.5/11.0). ACN was removed by vacuum centrifugation for 20 min at 45°C and eluates were subsequently desalted by C18 StageTipping prior to LC-MS/MS analysis on EASY-nLC1200 and Orbitrap Exporis 480 devices.

Raw files from SILAC-labeled ubiquitin remnant-enriched samples were analyzed using MaxQuant with multiplicity set to 2 (Lys0/Arg0 and Lys8/Arg10). The human UniProt reference proteome was used as the search database. Trypsin/P was specified as the enzyme, allowing up to two missed cleavages. Carbamidomethylation of cysteine was set as a fixed modification, while methionine oxidation, N-terminal acetylation, and GlyGly-lysine (K-ε-GG) were included as variable modifications. Peptide tolerance was 20 ppm for the first search and 4.5 ppm for the main search. Both peptide and protein FDR were controlled at 1%. SILAC quantification was performed using MaxQuant’s built-in normalization, and heavy/light ratios were calculated at the peptide level and collapsed to protein groups. Processed data were analyzed in R (4.4.2). The identified protein groups were filtered for potential contaminants, reverse-identified peptides, and localization probability (>75%). The *P*-values were calculated by a moderated *t* test using the limma package (Ritchie et al, 2015). For downstream analysis, only regulated ubiquitylation sites with *P*-value <0.05 were considered significant.

## Supplementary Material

Reviewer comments

## Data Availability

Source data for gels, Western blots, mass spectrometry, raw quantification data, microscopy and flow cytometry source data is available online via the following links: Source data for Main Figures are available online via Mendeley Data (Munro et al, 2026a). Source data for Supplementary Figures are available online via Mendeley Data (Munro et al, 2026b). Source data for mass spectrometry results is available via PRIDE with the accession number PXD082712: https://www.ebi.ac.uk/pride/archive/projects/PXD082712. Further information and request for resources and reagents should be directed to and will be fulfilled by the lead contact, Hemmo Meyer.
