# Supplementary material for: Induced degradation of Ufd1 reveals regulation of cohesin by the VCP/p97Ufd1-Npl4 complex
Source: Life Sci Alliance. 2026 Sep 3;9(11):e202603698. doi: 10.26508/lsa.202603698 (PMC13542637; doi:10.26508/lsa.202603698)
Supplement: Supplementary file 1 [file LSA-2026-03698_review_history.pdf]

Induced degradation of Ufd1 reveals regulation of cohesin by the VCP/p97Ufd1-Npl4 complex

Hemmo Meyer, Daniella Munro, Maike Reinders, Aikaterini Pravi, Johannes van den Boom, Pinki Gahlot, Ekaterina Isaakova, Caio Oliveira, Dominik Boos, and Petra Beli
DOI: https://doi.org/10.26508/lsa.202603698

Corresponding author(s): Hemmo Meyer, University of Duisburg-Essen

Review Timeline:

Submission Date:	2026-03-16
Editorial Decision:	2026-04-27
Revision Received:	2026-07-13
Editorial Decision:	2026-08-10
Revision Received:	2026-08-20
Accepted:	2026-08-26

Scientific Editor: Sarita Hebbar

Transaction Report:

(Note: With the exception of the correction of typographical or spelling errors that could be a source of ambiguity, letters and reports are not edited. The original formatting of letters and referee reports may not be reflected in this compilation.)

April 27, 2026

Re: Life Science Alliance manuscript #LSA-2026-03698-T

Prof. Hemmo H Meyer
Universitat Duisburg-Essen
Faculty of Biology
Institute of Molecular Biology 1
Campus Essen
Essen 45117
Germany

Dear Dr. Meyer,

Thank you for submitting your manuscript entitled "Induced degradation of Ufd1 reveals regulation of cohesin by the VCP/p97Ufd1-Npl4 complex" to Life Science Alliance. We apologise for the delay in communicating our decision due to editor availability issues.

Your manuscript was assessed by three expert reviewers, whose comments are appended to this letter. As you will note, the three reviewers are largely consistent in their opinion on the potential value of this work. Reviewer 1 and 2 have also positively commented on the execution of this study. That said, the reviewers have raised some concerns and highlighted aspects of that manuscript that could be strengthened. Overall, we invite you to submit a a revised version addressing the reviewers' concerns.

-We agree that you must resolve concerns on the effects of synchronisation (Reviewer 2, major points 5 and 7) and off-target effects (Reviewer 3, point 3) with some of the suggested experiments and relevant additions to the discussion.
-We concur that a revised ms must resolve all concerns of the reviewers related to appropriate sample size (Reviewers 1-2) and required quantification (Reviewers 1,2- Figure 2G, Reviewer 3 -Figure 3A).
-We bring your attention to several points of the reviewers to provide a comprehensive description of experiments and results (Reviewers 1, 3- Figure 4, Reviewer 2 - major point 1-ms data).

When submitting the revision, please include a letter addressing the reviewers' comments point by point. While a rebuttal must respond to all points in some form, additional experiments to resolve these points, other than indicated above, are not required.

I would be happy to discuss the revision in more detail via email or phone/videoconferencing. Please let me know which option you prefer, if any.

To upload the revised version of your manuscript, please log in to your account: https://lsa.msubmit.net/cgi-bin/main.plex

You will be guided to complete the submission of your revised manuscript and to fill in all necessary information. Please get in touch in case you do not know or remember your login name.

The typical timeframe for revisions is three months. Please note that papers are generally considered through only one revision cycle, so strong support from the referees on the revised version is needed for acceptance.

While you are revising your manuscript, please also attend to the below editorial points to help expedite the publication of your manuscript. Please direct any editorial questions to the journal office.

Thank you for this interesting contribution to Life Science Alliance. We hope that the comments below will prove constructive as your work progresses, and we are looking forward to receiving your revised manuscript.

Sincerely,

Sarita Hebbar, PhD
Scientific Editor
Life Science Alliance
http://www.lsajournal.org

A. THESE ITEMS ARE REQUIRED FOR REVISIONS

-- A letter addressing the reviewers' comments point by point.

-- An editable version of the final text (.DOC or .DOCX) is needed for copyediting (no PDFs).

-- High-resolution figure, supplementary figure and video files uploaded as individual files: See our detailed guidelines for preparing your production-ready images, https://www.life-science-alliance.org/authors

-- Summary blurb (enter in submission system): A short text summarizing in a single sentence the study (max. 200 characters including spaces). This text is used in conjunction with the titles of papers, hence should be informative and complementary to the title and running title. It should describe the context and significance of the findings for a general readership; it should be written in the present tense and refer to the work in the third person. Author names should not be mentioned.

-- By submitting a revision, you attest that you are aware of our payment policies found here: https://www.life-science-alliance.org/copyright-license-fee

B. MANUSCRIPT ORGANIZATION AND FORMATTING:

Full guidelines are available on our Instructions for Authors page, https://www.life-science-alliance.org/authors

We encourage our authors to provide original source data, particularly uncropped/-processed electrophoretic blots and spreadsheets for the main figures of the manuscript. If you would like to add source data, we would welcome one PDF/Excel-file per figure for this information. These files will be linked as supplementary "Source Data" files.

IMPORTANT: It is Life Science Alliance policy that if requested, original data images must be made available. Failure to provide original images upon request will result in unavoidable delays in publication. Please ensure that you have access to all original microscopy and blot data images before submitting your revision.

Reviewer #1 (Comments to the Authors (Required)):

This manuscript investigates the role of the p97 adapter Ufd1 on the ubiquitin-modified proteome. Using an induced-degradation approach, they identify proteins whose degree of ubiquitin modification changes when Ufd1 is down regulated, both in asynchronous cells as well as those that are treated with ionizing radiation. The mass spec based screen identified proteins that are known to be regulated by p97/Ufd1 and also identified Rad21 as more strongly ubiquitylated following depletion of Ufd1 under both conditions. They subsequently confirmed that down regulation of Ufd1 is associated with increased ubiquitylation of endogenous and exogenously expressed Rad21, which also correlated with loading of Rad21 on chromatin. Similar results were obtained with an inhibitor of p97. Using synchronized cells, the authors then show that Ufd1/p97 likely functions in S-phase to remove Rad21, and that failure to remove Rad21 results in replication-dependent damage.

Overall, I feel the experiments in the paper are well-executed and the conclusions are supported by the findings. The major limitation of the paper is that the results are correlative and thus it remains unclear that the observed defects in replication are directly due to the failure to extract Rad21 from chromatin. In their mass spec results, the authors indicate that Rad21 is ubiquitylated at K573. However, this site of ubiquitylation is not directly confirmed by independent experiments. For example, it would be interesting to know whether mutation of this site is sufficient to block ubiquitylation. If so, it would then be possible to use this mutant to determine whether it shows increased chromatin association and whether it is sufficient to induce a phenotype similar to Ufd1 inactivation. However, if there are multiple ubiquitylation sites involved, it could be challenging. At the least, the authors should discuss whether they have attempted such an approach.

The following minor issues should be addressed prior to publication:

-The text references Fig 4G, H but I do not see them:
"Replication-associated DNA damage induced by the degradation of Ufd1 was linked to cohesin dynamics because concomitant siRNA-mediated depletion of the cohesin loader NIPBL reduced the number of yH2AX foci caused by Ufd1 elimination during S phase (Fig. 4G,H)."

-What is reference to EV1X?

-Fig 2G should be quantitated and replicates shown

-Fig EV3A panels should be labeled. The legend is confusing, it should say that the two plots represent data from one of two independent experiments.

-For Fig 4 D I see 5 data points for each condition, but the figure legend states that it represents 5 technical replicates from 3 independent experiments. Is only one experiment shown?

-There is an error in the following sentence: In yeast, Rad21/Scc1 is ubiquitylated by Rsp5 for unloading by Cdc48 during replication (Frattini et al, 2017) consistent with our model for human cells model.

Reviewer #2 (Comments to the Authors (Required)):

In this manuscript Munro et al. investigate the effects of acutely depleting Ufd1, part of the Ufd1-Npl4 complex, which cooperates with p97 segregase. By doing ubiquiting profiling mass spectrometry, the authors show accumulation of ubiquitin at a plethora of proteins including the cohesin subunit Rad21. They show that Rad21 subunit persists on chromatin after Ufd1 depletion or p97 inhibition, specifically at the S-phase of the cell cycle. This accumulation drives DNA damage accumulation in a cohesin-dependent manner since depletion of NIPBL reduces this damage.

The manuscript is well written, and well discussed, proposing cautious conclusions for the less developed parts. Although the manuscript lacks mechanistic detail, the finding of a new way of regulating cohesin chromatin association through p97, and being independent of the know unloader WAPL is novel. However, I have a few questions/concerns that I would like them to address to strengthen the conclusions of the manuscript:

Minor:

1. The authors attribute the failure to endogenously tag Ufd1 with FKBP12 (F36V) to its essentiality, but literature is full of examples of essential proteins being able to be tagged with degron tags. Authors should rephrase or remove this sentence.

2. To show essentiality of Ufd1 un their genetically engineered cells, authors could perform viability assays in the presence of dTAG.

3. I believe Figure "EV1X" refers to figure EV1D. this one should change order and be EV1B. EV1B should now be EV1C, and EV1C should be EV1D.

4. Authors say that Rad21 interacts more strongly with p97 E578Q, but in my view this should be rephrased to indicate that the interaction is only visible with the mutants. No band can be appreciated in this blot at least, with p97-WT.

5. In this sentence: "Using a double thymidine block and a final release from the G1/S block for up to 6 h or 16 h, we synchronized cells for treatment and analysis specifically in G1 and S phases, respectively, and analysed RAD21-GFP in stable cells by microscopy (Fig. 3A,B)" G1 and S phase are the opposite. 6h should be S phase and 16h should be the following G1.

6. Authors do not refer in the text to Figures EV3A and B. Moreover, they should comment on the fact that synchronization in S-phase is mainly in the late-S phase stage, and this might bias the results. Performing an experiment with less time of release (3h) to see that results are not biased by this, is advised.

7. "consistent with our model for human cells model." The word "model" should be removed at the end of this sentence from the Discussion

Major:

1. Why do authors focus on proteins which are not top hits in the mass spectrometry results? A more detailed explanation would be appreciated. Also, a bit more rationale into why they decide to include IR in the experiments would be appreciated as well. In this regard they could also mention that cohesin is involved in DSB repair when naming its functions.

2. Not only Ufd1-depletion/p97 inhibition are antagonists of NIPBL, but also, in my view, these results suggest that NIBPL might not be a "loader" of cohesin but a loop extruder. The fact that cohesin can be loaded/accumulated on chromatin upon Ufd1-depletion/p97 inhibition in a siNIPBL background, suggests that in the absence of NIPBL cohesin might just be more sensitive to chromatin removal through p97 segregase. Authors should discuss this possibility.

3. The authors should stain in immunofluorescence experiments using p97i and Ufd1 degradation, for a different cohesin core subunit (SMC1 or SMC3) and see whether it is a one-subunit phenotype, or if as they assume, it also applies to the whole cohesin complex.

4. In Figure 2G, the statement that "the combination of WAPL depletion and Ufd1 degradation did not further increase the vermicelli", how is this evaluated? In % of cells with vermicelli? Is it a qualitative observation? Is there any control that authors can show of something adding up to vermicelli in WAPL-depleted cells, to know what they refer to? Authors should find a way to

quantify and use controls if available to support this statement.

5. In Figure 3, to avoid possible artefactual effects coming from cell synchronization, authors could do immunofluorescence of Rad21 in an asynchronous population and include EdU staining. Then they could separate EdU positive cells from negative cell and plot the intensity of Rad21 in EdU positive cells.

6. The authors should clearly indicate in the figure legends how many biological replicates they have for each experiment, and complete to at least 3 biological replicates for all the experiments in the manuscript.

7. Experiments in Figure 4 should be tested in asynchronous populations, to discard effects of synchronization, which completely arrests the replication forks and induces DNA damage signalling by itself. Analysis of phospho-H2AX foci in S-phase can be done by coupling the staining with EdU Click chemistry.

Reviewer #3 (Comments to the Authors (Required)):

The authors developed a dTAG-inducible system by introducing a Ufd1-FKBP12(F36V) fusion construct into a UFD1 knockout cell line to enable controlled depletion of Ufd1. Using this system in combination with ubiquitin-enrichment mass spectrometry, and with mild ionising radiation, they identified DNA damage responsive proteins that become increasingly ubiquitinated in the absence of Ufd1. Subsequent validation through immunoprecipitation and Western blotting, alongside experiments examining p97 deficiency, highlighted Rad21 (a core component of the cohesin complex) as a key regulatory target.
Further investigation in non-irradiated cells reveal that loss of Ufd1 or p97 leads to increased chromatin-bound Rad21. Notably, this effect is pronounced during S-phase but not in G1, indicating that Ufd1-p97 activity specifically regulates cohesin dynamics through Rad21 during DNA replication. Functionally, deficiency of Ufd1 or p97 during S-phase induces replication stress and DNA damage, as evidenced by reduced BrdU incorporation and elevated levels of phospho-CHK1, phospho-RPA32, and γH2AX. Importantly, these phenotypes can be partially reverted either by inhibiting DNA polymerases with aphidicolin or by reducing Rad21 loading by NIPBL knockdown.
Together, these findings support a model in which the p97-Ufd1 complex preserves genome stability during replication by allowing chromatin-bound Rad21 unloading.
This is a meaningful finding in the field of DNA replication and genome stability, as it links the p97-ubiquitin system to cohesion control during S-phase, especially in contexts where p97 inhibition is already known to be relevant as a cancer therapeutic target. The work is therefore relevant with potential downstream implications for disease research.
This reviewer, however, has minor comments that I believe require clarification:

1. The effect of Ufd1 depletion in G1-phase cells is not entirely convincing. In particular, the mean RAD21 signal in the control sample (Figure 3A) appears unusually elevated, which may reflect higher basal RAD21 protein and complicates interpretation of the observed differences. Could the authors comment on this? Moreover, this experiment appears to have been performed only once. Additional biological replicates demonstrating consistent results would be necessary to support this conclusion more robustly.

2. dTAG-CRBN induced degradation of Ufd1 is reported to trigger replication stress, as evidenced by increased phospho-CHK1, phospho-RPA2, and gammaH2AX (Figure 4B). However, it appears that dTAG-CRBN treatment is also associated with elevated levels of total CHK1. The authors should comment on this observation, as it may complicate the interpretation of CHK1 activation and raises the possibility of broader effects on protein stability or stress responses.

3. More generally, given that dTAG-CRBN engages the ubiquitin-proteasome system, it would be important to exclude compound-specific effects in this context. The inclusion of a negative control in Ufd1-proficient (wild-type) cells treated with dTAG-CRBN would help clarify whether the observed replication stress phenotypes are specifically due to Ufd1 degradation rather than off-target effects of dTAG-CRBN itself.

LSA-2026-03698-T resubmission

We thank the reviewers for the overall positive assessments. We believe we were able to address the main concerns raised by the reviewers: (1) We excluded an off-target effect of the dTAG compound on replication by testing parental cells (without the FKBP-tag on Ufd1). (2) We excluded synchronization artefacts on RAD21 localization by scoring cell cycle markers. (3) We repeated the key experiments to a total of at least 3 replica and completed statistics for the key experiments. Please see below for a point-by-point response also to the other comments raised.

Reviewer #1 (Comments to the Authors (Required)):
This manuscript investigates the role of the p97 adapter Ufd1 on the ubiquitin-modified proteome. Using an induced-degradation approach, they identify proteins whose degree of ubiquitin modification changes when Ufd1 is down regulated, both in asynchronous cells as well as those that are treated with ionizing radiation. The mass spec based screen identified proteins that are known to be regulated by p97/Ufd1 and also identified Rad21 as more strongly ubiquitylated following depletion of Ufd1 under both conditions. They subsequently confirmed that down regulation of Ufd1 is associated with increased ubiquitylation of endogenous and exogenously expressed Rad21, which also correlated with loading of Rad21 on chromatin. Similar results were obtained with an inhibitor of p97. Using synchronized cells, the authors then show that Ufd1/p97 likely functions in S-phase to remove Rad21, and that failure to remove Rad21 results in replication-dependent damage.
Overall, I feel the experiments in the paper are well-executed and the conclusions are supported by the findings. The major limitation of the paper is that the results are correlative and thus it remains unclear that the observed defects in replication are directly due to the failure to extract Rad21 from chromatin. In their mass spec results, the authors indicate that Rad21 is ubiquitylated at K573. However, this site of ubiquitylation is not directly confirmed by independent experiments. For example, it would be interesting to know whether mutation of this site is sufficient to block ubiquitylation. If so, it would then be possible to use this mutant to determine whether it shows increased chromatin association and whether it is sufficient to induce a phenotype similar to Ufd1 inactivation. However, if there are multiple ubiquitylation sites involved, it could be challenging. At the least, the authors should discuss whether they have attempted such an approach.

We thank the referee for the assessment. As for mutation of the ubiquitylation sites, we mutated the detected site K573, as well as groups of lysines predicted to be exposed to the surface of the cohesin complex, but did not see a decrease in overall ubiquitylation. This indicates that ubiquitin is conjugated to alternative sites without loss of function, as observed in so many other cases.

The following minor issues should be addressed prior to publication:
-The text references Fig 4G, H but I do not see them:
"Replication-associated DNA damage induced by the degradation of Ufd1 was linked to cohesin dynamics because concomitant siRNA-mediated depletion of the cohesin loader NIPBL reduced the number of yH2AX foci caused by Ufd1 elimination during S phase (Fig.4G,H)."

We apologize for this mistake. The corrected reference is Fig. 4E,F.

-What is reference to EV1X?

We apologize for this mistake. The reference is now EV1B showing all conditions extending on main Fig. 1E.

-Fig 2G should be quantitated and replicates shown

As requested, we quantified the effect of treatments on chromatin condensation ("vermicelli") using a categorization adapted from Wutz et al. (2017, PMID: 29217591). See Fig. 2G,H.

-Fig EV3A panels should be labeled. The legend is confusing, it should say that the two plots represent data from one of two independent experiments.

We corrected this accordingly.

-For Fig 4 D I see 5 data points for each condition, but the figure legend states that it represents 5 technical replicates from 3 independent experiments. Is only one experiment shown?

For clarity, we repeated these experiments. The figure now represents 5 biological replicates, confirming our conclusions. We updated the figure legend accordingly.

-There is an error in the following sentence: In yeast, Rad21/Scc1 is ubiquitylated by Rsp5 for unloading by Cdc48 during replication (Frattini et al, 2017) consistent with our model for human cells model.

We corrected the sentence.

Reviewer #2 (Comments to the Authors (Required)):
In this manuscript Munro et al. investigate the effects of acutely depleting Ufd1, part of the Ufd1-Npl4 complex, which cooperates with p97 segregase. By doing ubiquiting profiling mass spectrometry, the authors show accumulation of ubiquitin at a plethora of proteins including the cohesin subunit Rad21. They show that Rad21 subunit persists on chromatin after Ufd1 depletion or p97 inhibition, specifically at the S-phase of the cell cycle. This accumulation drives DNA damage accumulation in a cohesin-dependent manner since depletion of NIPBL reduces this damage.
The manuscript is well written, and well discussed, proposing cautious conclusions for the less developed parts. Although the manuscript lacks mechanistic detail, the finding of a new way of regulating cohesin chromatin association through p97, and being independent of the know unloader WAPL is novel.

However, I have a few questions/concerns that I would like them to address to strengthen the conclusions of the manuscript:

Minor:
1. The authors attribute the failure to endogenously tag Ufd1 with FKBP12 (F36V) to its essentiality, but literature is full of examples of essential proteins being able to be tagged with degron tags. Authors should rephrase or remove this sentence.

We agree. We removed the sentence, as requested.

2. To show essentiality of Ufd1 un their genetically engineered cells, authors could

perform viability assays in the presence of dTAG.

While this is a generally interesting question, we could not prioritize this point during the revision, because it does not directly contribute to the main line of conclusions.

3. I believe Figure "EV1X" refers to figure EV1D. this one should change order and be EV1B. EV1B should now be EV1C, and EV1C should be EV1D.

We apologize for the confusion. We correct the labeling accordingly.

4. Authors say that Rad21 interacts more strongly with p97 E578Q, but in my view this should be rephrased to indicate that the interaction is only visible with the mutants. No band can be appreciated in this blot at least, with p97-WT.

We rephrased accordingly.

5. In this sentence: "Using a double thymidine block and a final release from the G1/S block for up to 6 h or 16 h, we synchronized cells for treatment and analysis specifically in G1 and S phases, respectively, and analysed RAD21-GFP in stable cells by microscopy (Fig. 3A,B)" G1 and S phase are the opposite. 6h should be S phase and 16h should be the following G1.

We corrected that mistake. Thank you for pointing it out.

6. Authors do not refer in the text to Figures EV3A and B. Moreover, they should comment on the fact that synchronization in S-phase is mainly in the late-S phase stage, and this might bias the results. Performing an experiment with less time of release (3h) to see that results are not biased by this, is advised.

We now refer in the main text to EV3A and EV3B that verify the G1 and S synchronization efficiency. As for the S-phase synchronization, cells reach late-S phase by the end of the 6h chase, but treatments start during earlier phases of the S-phase and carry on until the 6h value as shown in the cartoon in Fig. 3B. In addition, we validated the S phase effects in parallel by categorization according to cell cycle markers rather than synchronization.

7. "consistent with our model for human cells model." The word "model" should be removed at the end of this sentence from the Discussion

We corrected the mistake. Thank you for pointing it out.

Major:

1. Why do authors focus on proteins which are not top hits in the mass spectrometry results? A more detailed explanation would be appreciated. Also, a bit more rationale into why they decide to include IR in the experiments would be appreciated as well. In this regard they could also mention that cohesin is involved in DSB repair when naming its functions.

The role of cohesion in DNA repair was mentioned already in the first manuscript version in the result sections. There, we also explain that we chose Rad21 because it emerged as a candidate also in unchallenged cells (no IR). We felt it was interesting to analyze a "housekeeping" role of p97, because DNA repair functions had been shown already in various cases. Nevertheless, the

inclusion of p97 candidates that respond to IR-induced DNA damage validates the approach and is a helpful contribution to the field. We therefore decided to include the IR condition also.

2. Not only Ufd1-depletion/p97 inhibition are antagonists of NIPBL, but also, in my view, these results suggest that NIBPL might not be a "loader" of cohesin but a loop extruder. The fact that cohesin can be loaded/accumulated on chromatin upon Ufd1-depletion/p97 inhibition in a siNIPBL background, suggests that in the absence of NIPBL cohesin might just be more sensitive to chromatin removal through p97 segregase. Authors should discuss this possibility.

We agree with the reviewer. However, as NIPBL is not the focus of this study, and we have no additional evidence to support this point, we did not want to lead the discussion in this direction.

3. The authors should stain in immunofluorescence experiments using p97i and Ufd1 degradation, for a different cohesin core subunit (SMC1 or SMC3) and see whether it is a one-subunit phenotype, or if as they assume, it also applies to the whole cohesin complex.

We tested the available antibodies for SMC1 and SMC3 but failed to establish reliable detection. Moreover, to our knowledge, the prevailing view is that RAD21 binds chromatin by forming a ring with SMC1/3 around DNA. We therefore do not feel that this approach justifies further delay of the revision.

4. In Figure 2G, the statement that "the combination of WAPL depletion and Ufd1 degradation did not further increase the vermicelli", how is this evaluated? In % of cells with vermicelli? Is it a qualitative observation? Is there any control that authors can show of something adding up to vermicelli in WAPL-depleted cells, to know what they refer to? Authors should find a way to quantify and use controls if available to support this statement.

As requested, we quantified the effect of treatments on chromatin condensation ("vermicelli") using a categorization similar to Wutz et al. (2017, PMID: 29217591) and also used PDS5A and PDS5B as positive controls for further stimulation of the WAPL effect. See Fig. 2G,H.

5. In Figure 3, to avoid possible artefactual effects coming from cell synchronization, authors could do immunofluorescence of Rad21 in an asynchronous population and include EdU staining. Then they could separate EdU positive cells from negative cell and plot the intensity of Rad21 in EdU positive cells.

We used the CyclinA2 marker to categorize the cell cycle state of cells. Consistent with our model, unsynchronized CyclinA2-positive (S/G2) cells showed a significant increase in RAD21 on chromatin, whereas CyclinA2-negative (G1-phase) cells did not (new Fig. EV3C,D). We used the EdU approach for the effect on gamma-H2AX (see point 7).

6. The authors should clearly indicate in the figure legends how many biological replicates they have for each experiment, and complete to at least 3 biological replicates for all the experiments in the manuscript.

We have now repeated all critical experiments at least 3 times as requested and indicated so in the figure legend.

7. Experiments in Figure 4 should be tested in asynchronous populations, to discard effects of synchronization, which completely arrests the replication forks and induces DNA damage

signalling by itself. Analysis of phospho-H2AX foci in Sphase can be done by coupling the staining with EdU Click chemistry.

As requested, we now analyzed unsynchronized cells and categorized them according to EdU incorporation (new Fig. EV4C,D). In line with our conclusions, we find that EdU-positive (S-phase) cells show an increase in gamma-H2AX foci upon dTAG addition compared to EdU-negative cells.

Reviewer #3 (Comments to the Authors (Required)):
The authors developed a dTAG-inducible system by introducing a Ufd1-FKBP12(F36V) fusion construct into a UFD1 knockout cell line to enable controlled depletion of Ufd1. Using this system in combination with ubiquitin-enrichment mass spectrometry, and with mild ionising radiation, they identified DNA damage responsive proteins that become increasingly ubiquitinated in the absence of Ufd1. Subsequent validation through immunoprecipitation and Western blotting, alongside experiments examining p97 deficiency, highlighted Rad21 (a core component of the cohesin complex) as a key regulatory target. Further investigation in non-irradiated cells reveal that loss of Ufd1 or p97 leads to increased chromatin-bound Rad21. Notably, this effect is pronounced during Sphase but not in G1, indicating that Ufd1-p97 activity specifically regulates cohesin dynamics through Rad21 during DNA replication. Functionally, deficiency of Ufd1 or p97 during S-phase induces replication stress and DNA damage, as evidenced by reduced BrdU incorporation and elevated levels of phospho-CHK1, phospho-RPA32, and γH2AX. Importantly, these phenotypes can be partially reverted either by inhibiting DNA polymerases with aphidicolin or by reducing Rad21 loading by NIPBL knockdown. Together, these findings support a model in which the p97-Ufd1 complex preserves genome stability during replication by allowing chromatin-bound Rad21 unloading.
This is a meaningful finding in the field of DNA replication and genome stability, as it links the p97-ubiquitin system to cohesion control during S-phase, especially in contexts where p97 inhibition is already known to be relevant as a cancer therapeutic target. The work is therefore relevant with potential downstream implications for disease research.

This reviewer, however, has minor comments that I believe require clarification:
1. The effect of Ufd1 depletion in G1-phase cells is not entirely convincing. In particular, the mean RAD21 signal in the control sample (Figure 3A) appears unusually elevated, which may reflect higher basal RAD21 protein and complicates interpretation of the observed differences. Could the authors comment on this? Moreover, this experiment appears to have been performed only once. Additional biological replicates demonstrating consistent results would be necessary to support this conclusion more robustly.

As requested, we have now repeated all critical experiments 3 times including the G1-phase analysis mentioned here. This confirmed no significant effect in G1 consistent with our conclusions.
As for the other point (unusual elevation), we are not sure exactly what the reviewer refers to. Fig. 3C (dTAG) shows an antibody staining, whereas Fig. 3D shows RAD21-GFP in stable cell lines - so intensities cannot be directly compared.

2. dTAG-CRBN induced degradation of Ufd1 is reported to trigger replication stress, as evidenced by increased phospho-CHK1, phospho-RPA2, and gammaH2AX (Figure 4B). However, it appears that dTAG-CRBN treatment is also associated with elevated levels of total CHK1. The authors should comment on this observation, as it may complicate the interpretation

of CHK1 activation and raises the possibility of broader effects on protein stability or stress responses.

We acknowledge the increase of CHK1 protein level in the western blot which may be due to a possible involvement of Ufd1 in constitutive CHK1 turnover. However, the increase of phosphorylation is considerable higher (roughly fourfold increase compared to 50-100% increase in protein levels when quantified by densitometry).

3. More generally, given that dTAG-CRBN engages the ubiquitin-proteasome system, it would be important to exclude compound-specific effects in this context. The inclusion of a negative control in Ufd1-proficient (wild-type) cells treated with dTAG-CRBN would help clarify whether the observed replication stress phenotypes are specifically due to Ufd1 degradation rather than off-target effects of dTAGCRBN itself.
As requested, we now tested the dTAG on parental HeLa cells (without FKBP-tag fused to Ufd1) and see no significant reduction on DNA synthesis (new Fig. EV4B).

August 11, 2026

RE: Life Science Alliance Manuscript #LSA-2026-03698-TR

Prof. Hemmo Meyer
University of Duisburg-Essen
Faculty of Biology
Institute of Molecular Biology 1
Campus Essen
Essen 45117
Germany

Dear Dr. Meyer,

Thank you for submitting your revised manuscript entitled "Induced degradation of Ufd1 reveals regulation of cohesin by the VCP/p97Ufd1-Npl4 complex".

Your revised manuscript was evaluated by all the original reviewers whose comments are appended below. As you will read, the reviewers have acknowledged that your revised manuscript is strengthened having largely addressed their previous concerns. Reviewer 1 has offered a minor suggestion that we leave to your choice to incorporate.

In line with the reviewers' overall evaluation, we would be happy to publish your paper in Life Science Alliance pending resolution of final revisions necessary to meet our formatting guidelines.

MANUSCRIPT ORGANIZATION AND FORMATTING:

To avoid unnecessary delays in the acceptance and publication of your paper, please read the following information carefully. Full guidelines are available on our Instructions for Authors page, https://www.life-science-alliance.org/authors

Submission of a paper that does not conform to Life Science Alliance guidelines will delay the acceptance of your manuscript.

-Please provide primer sequence information used to amplify human HUS1 and humanMCM7 were amplified from HeLa cDNA (line 266).
-Please expand on method for knockout of the UFD1 gene using CRISPR Cas9 technology with guide RNA sequence information (line 275).
-Thank you for providing your statement on data availability. You can remove the references to the EV Figures since all the supplementary figures have been uploaded to the system.
Please refer to our guidelines on data deposition (https://www.life-science-alliance.org/manuscript-prep#datadepot) and data availability (https://www.life-science-alliance.org/editorial-policies#data-sharing) and accordingly modify your data availability statement to reflect the public database and accession code for mass spec data.
-Methods section should be labeled as 'Materials and methods'.
-LSA allows supplementary figures, but no EV Figures,Supplementary figures use the system.supplementary Figure S1 etc.; please update your callouts for the Supplementary Figures in the manuscript Fig EV1A=Fig S1A etc.
-Please add the X and Bluesky handles of your host institute/organization, as well as your own, and/or one of the authors, in our system.
-Please be sure that the authorship listing and order is correct.

We encourage our authors to provide original source data, particularly uncropped/-processed electrophoretic blots and spreadsheets for the main figures of the manuscript. If you would like to add source data, we would welcome one PDF/Excel-file per figure for this information. These files will be linked as supplementary "Source Data" files.

It is Life Science Alliance policy that if requested, original data images must be made available to the editors. Failure to provide original images upon request will result in unavoidable delays in publication. Please ensure that you have access to all original data images prior to final submission.

If you are planning a press release on your work, please inform us immediately to allow informing our production team and scheduling a release date.

We welcome submissions of potential cover images for the issue of LSA in which your work would appear. If you have high quality images associated with this work, please feel free to email these, with a caption, to the journal office.

LSA encourages authors to provide a 30-60 second video where the study is briefly explained. These videos will be appear embedded with the manuscript online at Life Science Alliance, and on social media to promote the published paper and authors (for examples, see https://docs.google.com/document/d/1-UWCfbE4pGcDdcgzcmiuJl2XMBJnxKYeqRvLLrLSo8s/edit?usp=sharing). Corresponding or first-authors are welcome to submit the video. Please submit only one video per manuscript. The video can be emailed to contact@life-science-alliance.org

FINAL FILES:

To upload the final version of your manuscript, please log in to your account: https://lsa.msubmit.net/cgi-bin/main.plex
You will be guided to complete the submission of your revised manuscript and to fill in all necessary information. Please get in touch in case you do not know or remember your login name.

The following items are required for acceptance.

-- An editable version of the final text (.DOC or .DOCX) is needed for copyediting (no PDFs).

-- High-resolution figure, supplementary figure and video files uploaded as individual files: See our detailed guidelines for preparing your production-ready images, https://www.life-science-alliance.org/authors

-- Summary blurb (enter in submission system): A short text summarizing in a single sentence the study (max. 200 characters including spaces). This text is used in conjunction with the titles of papers, hence should be informative and complementary to the title. It should describe the context and significance of the findings for a general readership; it should be written in the present tense and refer to the work in the third person. Author names should not be mentioned.

The license to publish form must be signed before your manuscript can be sent to production. A link to the license to publish form will be available to the corresponding author only. Please take a moment to check your funder requirements.

Reviews, decision letters, and point-by-point responses associated with peer-review at Life Science Alliance will be published, alongside the manuscript. If you do want to opt out of having the reviewer reports and your point-by-point responses displayed, please let us know immediately.

Thank you for your attention to these final processing requirements. Please revise and format the manuscript and upload materials as soon as you are able.

Thank you for this interesting contribution to the literature. We look forward to publishing your paper in Life Science Alliance.

Sincerely,

Sarita Hebbar, PhD
Scientific Editor
Life Science Alliance
http://www.lsajournal.org

Reviewer #1 (Comments to the Authors (Required)):

The authors have responded to the points I raised in the review and I believe the manuscript is now suitable for publication.

Reviewer #2 (Comments to the Authors (Required)):

The authors have addressed most of my concerns. Those that could not be addressed do not affect the main conclusions of the manuscript.

Reviewer #3 (Comments to the Authors (Required)):

I thank the authors for their constructive responses to my comments. The revised manuscript has been strengthened and my concerns have been satisfactorily addressed. I provide only one minor suggestion that may further improve the presentation of the data.
1. Regarding the elevated RAD21 signal in the control sample, I was referring to Figure 3C (apologies for incorrectly referring to Figure 3A in the previous revision). Compared with the control samples shown in Figures 2B, 2D, and 2E, the basal nuclear

RAD21 signal in the original Figure 3C appeared noticeably higher, resulting in relatively small differences between control and dTAG-CRBN-treated cells. In the revised manuscript, the experiment now comprises four biological replicates, and both control and treated samples display lower and more comparable RAD21 signal intensities. The absence of a significant difference is therefore better supported.

2. Following this clarification, it would be helpful if the authors could include the densitometric quantification of the total CHK1 and phospho-CHK1 immunoblots to support their claim that the increase in phospho-CHK1 phosphorylation substantially exceeds the increase in total CHK1 levels.

3. The inclusion of the additional control experiment in parental, Ufd1-proficient cells demonstrates that dTAG-CRBN treatment alone does not significantly impair DNA synthesis, thereby strengthening the conclusion that the observed replication stress phenotype is not an off-target effect of the degrader, but a consequence of Ufd1 degradation.

LSA-2026-03698-TR resubmission

Reviewer #1 (Comments to the Authors (Required)):

The authors have responded to the points I raised in the review and I believe the manuscript is now suitable for publication.

Reviewer #2 (Comments to the Authors (Required)):

The authors have addressed most of my concerns. Those that could not be addressed do not affect the main conclusions of the manuscript.

Reviewer #3 (Comments to the Authors (Required)):

I thank the authors for their constructive responses to my comments. The revised manuscript has been strengthened and my concerns have been satisfactorily addressed. I provide only one minor suggestion that may further improve the presentation of the data.

1. Regarding the elevated RAD21 signal in the control sample, I was referring to Figure 3C (apologies for incorrectly referring to Figure 3A in the previous revision). Compared with the control samples shown in Figures 2B, 2D, and 2E, the basal nuclear RAD21 signal in the original Figure 3C appeared noticeably higher, resulting in relatively small differences between control and dTAG-CRBN-treated cells. In the revised manuscript, the experiment now comprises four biological replicates, and both control and treated samples display lower and more comparable RAD21 signal intensities. The absence of a significant difference is therefore better supported.
2. Following this clarification, it would be helpful if the authors could include the densitometric quantification of the total CHK1 and phospho-CHK1 immunoblots to support their claim that the increase in phospho-CHK1 phosphorylation substantially exceeds the increase in total CHK1 levels.
3. The inclusion of the additional control experiment in parental, Ufd1-proficient cells demonstrates that dTAG-CRBN treatment alone does not significantly impair DNA synthesis, thereby strengthening the conclusion that the observed replication stress phenotype is not an off-target effect of the degrader, but a consequence of Ufd1 degradation.

We thank the reviewers for the positive assessments. Regarding reviewer #3 point 2, we have now included a relative quantification of total CHK1 versus phospho-CHK1 intensities in Fig. 4B, which illustrates the increase in CHK1 phosphorylation beyond the increase in total CHK1, consistent with our conclusions.

August 26, 2026

RE: Life Science Alliance Manuscript #LSA-2026-03698-TRR

Prof. Hemmo Meyer
University of Duisburg-Essen
Faculty of Biology
Institute of Molecular Biology 1
Campus Essen
Essen 45117
Germany

Dear Dr. Meyer,

Thank you for submitting your Research Article entitled "Induced degradation of Ufd1 reveals regulation of cohesin by the VCP/p97Ufd1-Npl4 complex" and responding promptly to our last formatting request on your statement on data availability.

It is a pleasure to let you know that your manuscript is now accepted for publication in Life Science Alliance. Congratulations on this interesting work.

The final published version of your manuscript will be deposited by us to PubMed Central upon publication.

Your manuscript will now progress through copyediting and proofing. It is journal policy that authors provide original data upon request.

Your article will publish open access upon publication under a CC-BY license.

Reviews, decision letters, and point-by-point responses associated with peer-review at Life Science Alliance will be published, alongside the manuscript. If you do want to opt out of having the reviewer reports and your point-by-point responses displayed, please let us know immediately.

IMPORTANT: If you will be unreachable at any time, please provide us with the email address of an alternate author. Failure to respond to routine queries may lead to unavoidable delays in publication.

Scheduling details will be available from our production department. You will receive proofs shortly before the publication date. Only essential corrections can be made at the proof stage so if there are any minor final changes you wish to make to the manuscript, please let the journal office know now.

DISTRIBUTION OF MATERIALS:
Authors are required to distribute freely any materials used in experiments published in Life Science Alliance. Authors are encouraged to deposit materials used in their studies to the appropriate repositories for distribution to researchers.

You can contact the journal office with any questions, contact@life-science-alliance.org

Again, congratulations on a very nice paper. I hope you found the review process to be constructive and are pleased with how the manuscript was handled editorially. We look forward to future exciting submissions from your lab.

Sincerely,

Sarita Hebbar, PhD
Scientific Editor
Life Science Alliance
http://www.lsajournal.org
